# Supplementary material for: Population genomics and geographic dispersal in Chagas disease vectors: Landscape drivers and evidence of possible adaptation to the domestic setting
Source: PLoS Genet. 2022 Feb 4;18(2):e1010019. doi: 10.1371/journal.pgen.1010019 (PMC8849464; doi:10.1371/journal.pgen.1010019)
Supplement: S1 Methods — (PDF) [file pgen.1010019.s001.pdf]

S1 Methods. Data decontamination in *Rhodnius ecuadoriensis* sequenced reads.

Our decontamination pipeline (Fig 1) started by mapping *R. ecuadoriensis* reads to the *R. prolixus* annotated genome [1] using the Burrows- Wheeler algorithm [2] (BWA) implemented in DeconSeq standalone v4.3 [3]. We parametrised the DeconSeq programme by running it on a subset of *R. prolixus* high-quality 2b-RAD reads against the *R. prolixus* reference genome and varying the alignment identity threshold (-i) at 75, 85 and 95. After this trial, the DeconSeq run on the *R. ecuadoriensis* reads was set to 85. *R. ecuadoriensis* reads that mapped to the *R. prolixus* reference genome were kept and unmapped reads were further decontaminated. In the next step, *R. ecuadoriensis* reads that did not map to the *R. prolixus* genome were classified based on a bacterial, archaeal and viral genome database in the Kraken [4] programme using its taxonomy classification algorithm. *R. ecuadoriensis* reads classified as bacterial, archaeal and viral genomes by Kraken were discarded from the pipeline and those unclassified continued into the next decontamination step. Subsequent decontamination steps involved running again the DeconSeq programme on the unclassified *R. ecuadoriensis* reads, first, against the *T. cruzi* I Sylvio X10/1 genome [5] obtained from TriTrypDB.org genome database[6] and, then, against the human reference genome build 38 obtained from the National Centre for Biotechnology Information (NCBI) FTP server (ftp://ftp.ncbi.nih.gov/genomes/H_sapiens/Assembled_chromosomes/seq/). *R. ecuadoriensis* reads that mapped to the *T. cruzi* and Human genomes were discarded from the pipeline, whereas the rest that did not map were kept. Finally, those *R. ecuadoriensis* reads that did not map to any organisms above were merged with the reads that mapped to the *R. prolixus* genome at the beginning of the pipeline. At the end of the decontamination process, samples below a threshold of 100,000 decontaminated reads (hereafter called clean reads) were not included in the genotyping pipeline.

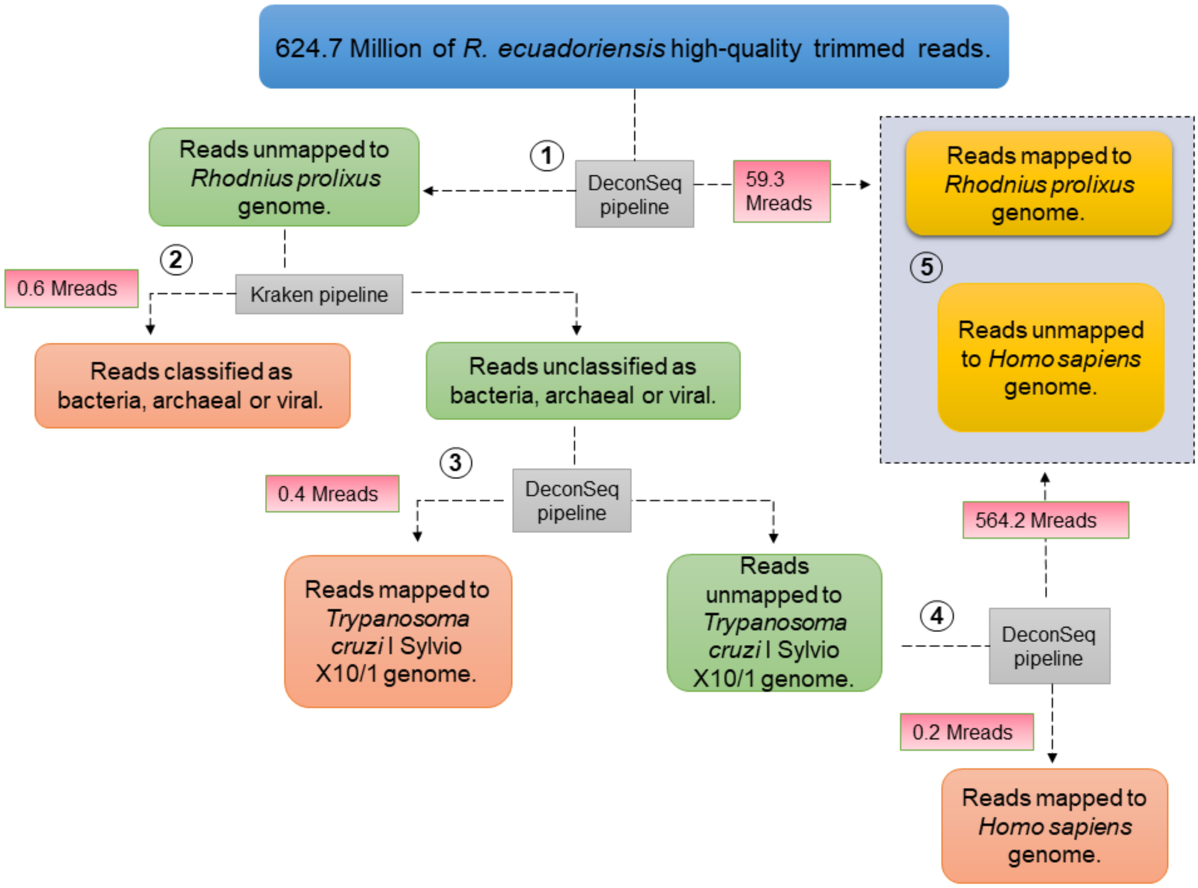

Fig 1. *R. ecuadoriensis* high-quality trimmed reads decontamination pipeline. Diagram shows the steps carried out to remove contaminant sequences from the *R. ecuadoriensis* high-quality trimmed reads. 1. *R. ecuadoriensis* reads were mapped against *R. prolixus* genome using DeconSeq programme [3] in which mapped reads (yellow box) were kept and unmapped reads (green box) were further decontaminated. 2. Unmapped reads (green box) were classified based on a bacterium, archaeal and viral database of the Kraken programme [4] and the resulting classified reads (red box) were discarded whereas unclassified reads (green box) were passed onto next steps. The "free from bacteria, archaeal and viral" reads (green box) were mapped against *T. cruzi* (3) and human genomes (4) using the DeconSeq programme. In both cases, mapped reads (Red boxes) were discarded from the pipeline and the reads that did not map to neither of genomes (yellow box) were merged (5) to the *R. ecuadoriensis* reads mapped to the *R. prolixus* genome at the beginning of the pipeline (dashed purple box).

References.

1. Mesquita RD, Vionette-Amaral RJ, Lowenberger C, Rivera-Pomar R, Monteiro FA, Minx P, et al. Genome of *Rhodnius prolixus* , an insect vector of Chagas disease, reveals unique adaptations to hematophagy and parasite infection. Proc Natl Acad Sci. 2015;112: 14936–14941. doi:10.1073/pnas.1506226112
2. Li H, Durbin R. Fast and accurate long-read alignment with Burrows–Wheeler transform. Bioinformatics. 2010;26: 589–595. doi:10.1093/bioinformatics/btp698
3. Schmieder R, Edwards R. Fast Identification and Removal of Sequence Contamination from Genomic and Metagenomic Datasets. Rodriguez-Valera F, editor. PLoS One. 2011;6: e17288. doi:10.1371/journal.pone.0017288
4. Wood DE, Salzberg SL. Kraken: ultrafast metagenomic sequence classification using exact alignments. Genome Biol. 2014;15: R46. doi:10.1186/gb-2014-15-3-r46
5. Franzén O, Ochaya S, Sherwood E, Lewis MD, Llewellyn MS, Miles MA, et al. Shotgun Sequencing Analysis of Trypanosoma cruzi I Sylvio X10/1 and Comparison with T. cruzi VI CL Brener. Bates PA, editor. PLoS Negl Trop Dis. 2011;5: e984. doi:10.1371/journal.pntd.0000984
6. Luchtan M, Warade C, Weatherly DB, Degrave WM, Tarleton RL, Kissinger JC. TcruziDB: an integrated Trypanosoma cruzi genome resource. Nucleic Acids Res. 2004;32: 344D – 346. doi:10.1093/nar/gkh049
