# Supplementary material for: Population genomics and geographic dispersal in Chagas disease vectors: Landscape drivers and evidence of possible adaptation to the domestic setting
Source: PLoS Genet. 2022 Feb 4;18(2):e1010019. doi: 10.1371/journal.pgen.1010019 (PMC8849464; doi:10.1371/journal.pgen.1010019)
Supplement: S2 Methods — (PDF) [file pgen.1010019.s002.pdf]

S2 Methods. Optimisation of genotyping strategy.

Optimisation pipeline. It consisted in testing eight different combinations (Table 1) of the STACKS [1] DENOVO_MAP.PL programme main control parameters (-m, -M, -n and -N) and selecting the combination resulting in the highest number of polymorphic loci but maintaining a low percentage of missing data and error rate [2,3]. To increase computational speed, we limited genotyping to a representative sample (n = 81) of the complete dataset consisting of 75 *R. ecuadoriensis* samples from six communities (SF, CG, BR, HY, GL, CQ) widely distributed in Loja and one from Manabí, and six *R. prolixus* samples. We used the STACKS parameter combination in our previous *R. ecuadoriensis* genotyping study [4] as a reference for the first run (Table 1 setting ID m5). In subsequently runs (Table 1 setting ID m4, m3, m2, M3, n0, n2 and n3), we varied one of the main control parameters at a time and kept the reference combination values for the rest of the parameters. Three values of parameter -m (4,3 and 2), one of parameter -M (3) and three of parameter - n (0, 2 and 3) were evaluated. As a reminder, parameters -m and -M are likely to have the most impact on the number of loci yielded. Setting -m too high will drop out true alleles of the pipeline, whereas low values will tend to increase potential sequencing error. Setting -M too low will increase the probability of missing SNPs, whereas low values will allow enough mismatches to build nonsensical biologically incorrect alleles. After -m and M have perfectively assembled loci, then -N recovers a set of “secondary” putative loci that did not achieved enough depth with -m, these set of secondary alleles aid the SNP calling model in detecting polymorphisms as they increase read depth. Finally, -n will look into the population catalog, consensus of all discovered loci, and it will try to merge loci across samples. Setting -n too will allow representation of independent loci in the catalog that in reality are the same locus [1].

Table 1 Description of the STACKS main control parameters and combinations tested for de novo assembly optimisation. -m is the minimum number of raw reads required to form a stack (a putative allele) which is comparable to the minimum depth of coverage. - M is Number of mismatches allowed between stacks (putative alleles) to merge them into a putative locus which is comparable to the number of nucleotide mismatches allowed. -n is the number of mismatches allowed between stacks (putative loci) during construction of the catalog that contains all loci and alleles of the population. -N is the number of mismatches allowed to align secondary reads (reads that did not form stacks) to assemble putative loci to increase locus depth. -alpha is the significance level to call a heterozygote or homozygote. -bound_low and -bound_high set the bounded SNP calling model for identifying a SNP and estimating the error rate at that SNP. -r is the percentage of individuals that must possess a particular locus for it to be included in calculation of population-level statistics. -min_maf specify the minor allele frequency for a particular locus, alleles occurring below this frequency are discarded. Further details in refs. [1–4].

Setting ID	*-m*	*-M*	*-n*	*-N*	*-alpha*	*-bound_low*	*-bound_high*	*-r*	*-min_maf*
*m5**	**5**	**2**	**1**	**4**	**0.01**	**0**	**0.05**	**0.8**	**0.01**
m4	**4**	2	1	4	0.01	0	0.05	0.8	0.01
m3	**3**	2	1	4	0.01	0	0.05	0.8	0.01
m2	**2**	2	1	4	0.01	0	0.05	0.8	0.01
M3	5	**3**	1	5	0.01	0	0.05	0.8	0.01
n0	5	2	**0**	4	0.01	0	0.05	0.8	0.01
n2	5	2	**2**	4	0.01	0	0.05	0.8	0.01
n3	5	2	**3**	4	0.01	0	0.05	0.8	0.01

* STACKS parameter combination used in [4]

References.

1. Catchen J, Hohenlohe PA, Bassham S, Amores A, Cresko WA. Stacks: an analysis tool set for population genomics. Mol Ecol. 2013;22: 3124–40. doi:10.1111/mec.12354
2. Mastretta-Yanes A, Arrigo N, Alvarez N, Jorgensen TH, Piñero D, Emerson BC. Restriction site-associated DNA sequencing, genotyping error estimation and de novo assembly optimization for population genetic inference. Mol Ecol Resour. 2015;15: 28–41. doi:10.1111/1755-0998.12291
3. Paris JR, Stevens JR, Catchen JM. Lost in parameter space: a road map for stacks. Johnston S, editor. Methods Ecol Evol. 2017;8: 1360–1373. doi:10.1111/2041-210X.12775
4. Hernandez-Castro LE, Paterno M, Villacís AG, Andersson B, Costales JA, De Noia M, et al. 2b-RAD genotyping for population genomic studies of Chagas disease vectors: Rhodnius ecuadoriensis in Ecuador. Ghedin E, editor. PLoS Negl Trop Dis. 2017;11: e0005710. doi:10.1371/journal.pntd.0005710
