# Supplementary material for: Population genomics and geographic dispersal in Chagas disease vectors: Landscape drivers and evidence of possible adaptation to the domestic setting
Source: PLoS Genet. 2022 Feb 4;18(2):e1010019. doi: 10.1371/journal.pgen.1010019 (PMC8849464; doi:10.1371/journal.pgen.1010019)
Supplement: S3 Methods — (PDF) [file pgen.1010019.s003.pdf]

S3 Methods. Genomic scans in domestic and wild *Rhodnius ecuadoriensis*.

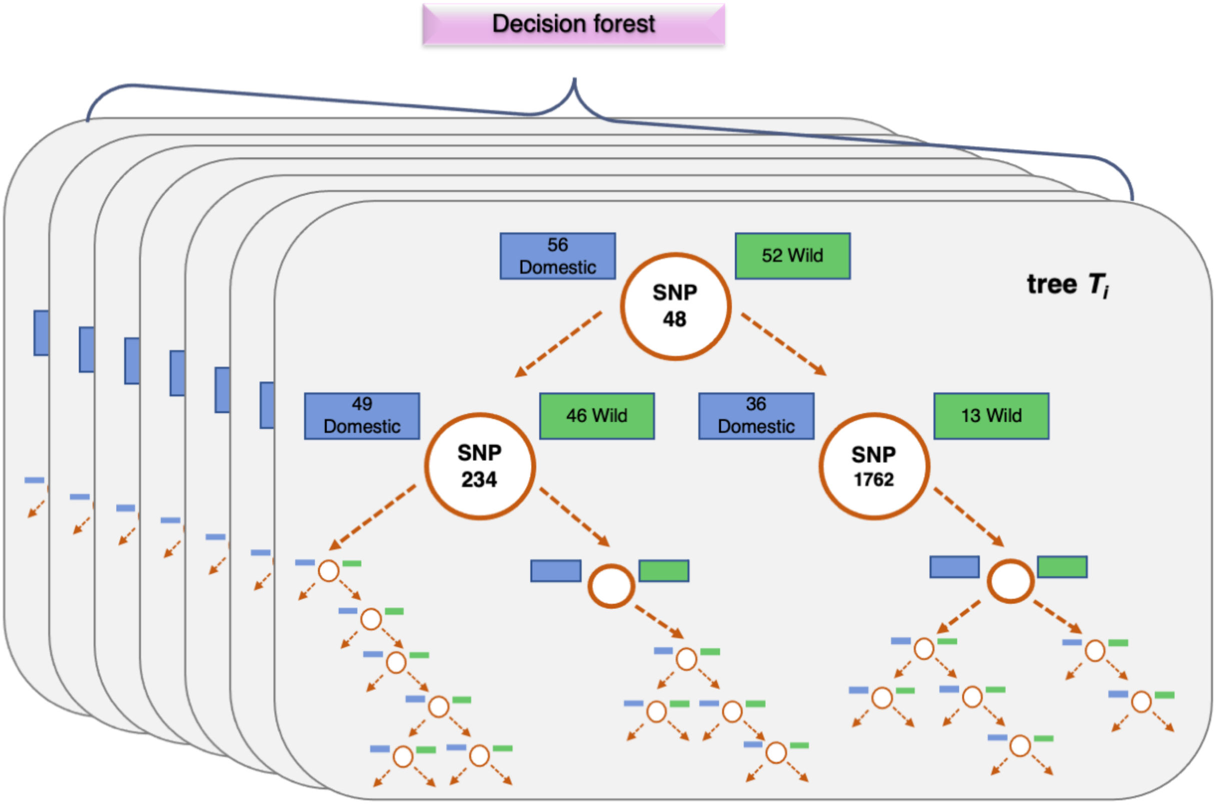

Fig 1. Random Forest procedure. The algorithm starts training by building a n number of decision trees, a forest, with 66.6% of the samples [1]. Each decision tree attempts to find the best SNPs that explain most of the within group variation (e.g. domestic and wild groups) until it is fully grown. Once trained, it compares the results with the remaining 33.3% of the samples and the misclassification of samples, or our-of-bag error rate, is used to find the SNPs with the best classification power. Boxes are colour-coded to represent domestic (blue) and wild (green) samples. SNPs and SNPs identification number are shown inside white circles. Each grey rectangle large box represents a decision tree within a large decision forest.

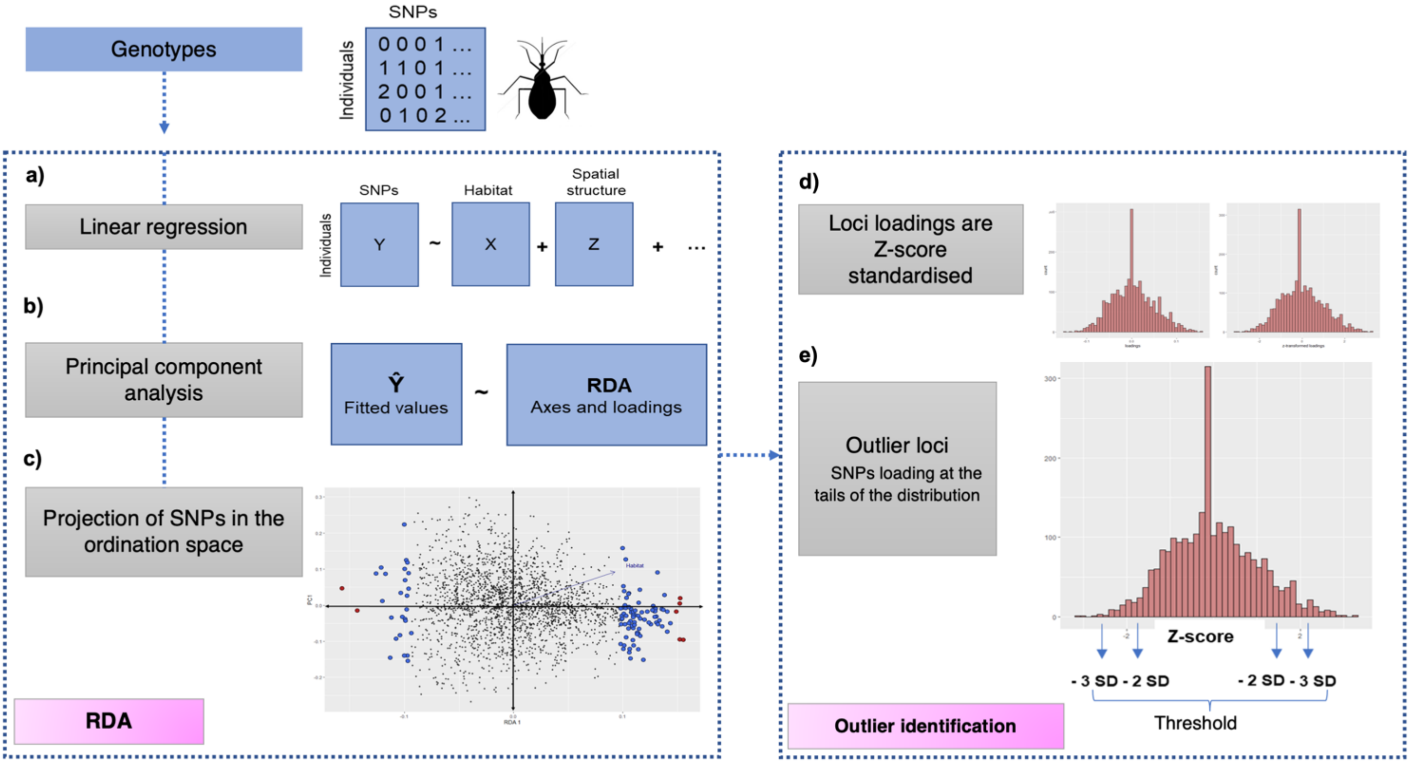

Fig 2. Step-by-step process of redundancy analysis (RDA). The RDA performs, **a**, multivariate regression between a response matrix of genotypes, Y, and a matrix of explanatory variables, X [2]. In our case, we added a conditional term to control for spatial structure, Z, based on the axes on individual principal coordinates of each sample. Then, a principal components analysis is carried out, **b**, on the matrix of fitted values, Ŷ, estimated from the regression on each locus. **c**, Relationship between SNPs coordinates and RDA axis can be explored in the ordination space. To identify outlier loci, **d**, SNPs loadings are Z-score standardised and, **e**, loci that fall at the tails of the distribution at a determine threshold (±2 SD or ±3 SD) are considered as outlier.

References.

1. Brieuc MSO, Waters CD, Drinan DP, Naish KA. A practical introduction to Random Forest for genetic association studies in ecology and evolution. Mol Ecol Resour. 2018;18: 755–766. doi:10.1111/1755-0998.12773
2. Capblancq T, Luu K, Blum MGB, Bazin E. Evaluation of redundancy analysis to identify signatures of local adaptation. Mol Ecol Resour. 2018;18: 1223–1233. doi:10.1111/1755-0998.12906
