# Supplementary material for: Population genomics and geographic dispersal in Chagas disease vectors: Landscape drivers and evidence of possible adaptation to the domestic setting
Source: PLoS Genet. 2022 Feb 4;18(2):e1010019. doi: 10.1371/journal.pgen.1010019 (PMC8849464; doi:10.1371/journal.pgen.1010019)
Supplement: S4 Methods — (PDF) [file pgen.1010019.s004.pdf]

S4 Methods. Landscape genomics mixed modelling framework on arthropod vectors.

Landscape genomics [1] can complement our understanding of arthropod vectors dispersal dynamics by estimating functional connectivity [2], the level at which the landscape facilitates or impedes their movement from, and to, different habitat patches [3]. This approach involves several stages starting with defining a clear research question which is typically related to investigating landscape arrangement effects on gene flow and/or local adaptation [4]. At this stage, the extent and resolution of an a priori landscape model is hypothesised based on empirical knowledge of factors (e.g. relief, temperature, land cover etc) likely affecting gene flow of the species of interest. Alternatively, simulation studies could help with specifying a landscape model and supporting spatial and genetic sampling design [5]. Then, pairwise population genetic distance (GD) information is obtained by sampling and genotyping individuals (or populations) across the defined landscape model at different gradients and time periods: both spatial and temporal scales are important considerations in a landscape genetic study [4,6]. Subsequently, the hypothetical landscape model is parametrised in order to obtain a resistance surface (spatial representation of a species movement constraints at each grid cell on a digital layer) from which pairwise population effective distances (ED) estimates of landscape connectivity (comparable to pairwise genetic data) are calculated using least-cost path, commute distance or circuit theory methods [7]. Finally, pairwise population genetic and landscape connectivity distances among points are correlated by computing available statistical approaches [8] such as mantel tests, regression, Bayesian inference, ordination techniques, and more recently, mixed effect models with maximum likelihood population effects parametrisation [9].

ResistanceGA optimisation process. The ResistanceGA algorithm (Fig 1) considers an initial population made of each raster surface as individuals experiencing evolution over generations. Raster surfaces resistance weights are the parameters to be optimised (Fig 1A). Fitness of a set of parameters is evaluated through an objective function which trains the genetic algorithm (Fig 1B). The training is made every time pairwise genetic (response) and effective (predictor) distances are regressed using a linear mixed-effects models with maximum likelihood population effect (MLPE) parametrization. Effective distances are calculated from the individual parameters being evaluated at each iteration (see below details on effective distances calculation). In our case, the machine learning objective function was based on the MLPE model which used the log-likelihood to quantify model performance. In machine learning, the objective function provides a way to assess parameters fitness, the most feasible parameters will have a better chance to be the "parents" of the next generation, but mutation will control stochasticity in the process [10,11]. MLPE regression overcomes the non-independence problem attached to pairwise data [12] and has been shown to be the best model selection method in landscape genetic studies (see refs. [13,14]). Different combinations of parameters (also seen as "genotypes" within an evolutionary context) in each individual are evaluated iteratively every generation and the fittest individuals (selection) and their parameters are passed over (crossover) into the next generation with top parameters slightly changing at random (mutation). The whole optimisation is repeated until raster surfaces and parameters (model fit) cannot be improved for several generations (convergence check - Fig 1C).

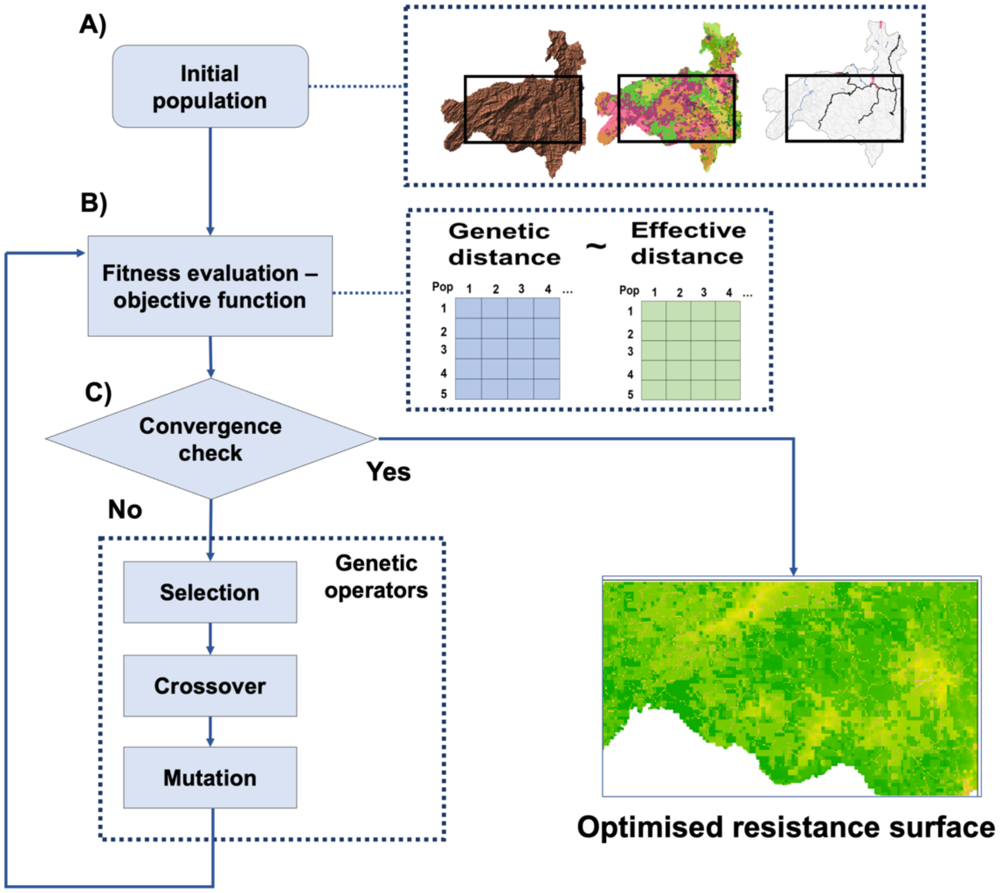

Fig 1. Schematic description of resistance surface optimisation in ResistanceGA. A, The process starts with an initial population made of raster surfaces and their weighted parameters. **B,** An objective function is used to train in the GA algorithm. The objective function attempts to solve a linear mixed effects model with MLPE parametrisation that regress genetic and effective distances matrices. **C,** If the model fit cannot be improved, the process terminates, otherwise it continues finding the fittest individuals and weighted parameters using an evolvability process. This process selects (selection) the best individuals, "parents", and reproduces (crossover) them to create a new generation with the best weighted parameters but slightly changed at random (mutation). The output of the ResistanceGA process is an optimised resistance surface with the optimal parameters solution. Source maps: www.usgs.gov/centers/eros/science/usgs-eros-archive-digital-elevation-global-multi-resolution-terrain-elevation, www.usgs.gov/media/images/south-america-land-cover-characteristics-data-base-version-20 and dataportaal.pbl.nl/downloads/GRIP4/GRIP4_Region2_vector_shp.zip.

In our analysis, effective distances (represented as commute distances) between sample sites locations (nodes) were calculated through random-walk commute time algorithm[15] on our optimised surface (Fig 2A) using the commuteDistance function implemented in the

gdistance [16] R package. Briefly, commute distance (Fig 2B) can be defined as the expected average time a random walker travels, back-and-forth, between two nodes through a set of paths, which is analogous to an electrical circuit. Commute distance is an equivalent to resistance distance, but the latter accounts for passage cost and availability of alternative paths. Resistance distances can also be calculated in ResistanceGA by calling the circuit theory algorithm [17,18] (Fig 2C) of CIRCUITSCAPE v5 [19] software, however, computation is slightly slower than using commuteDistance.

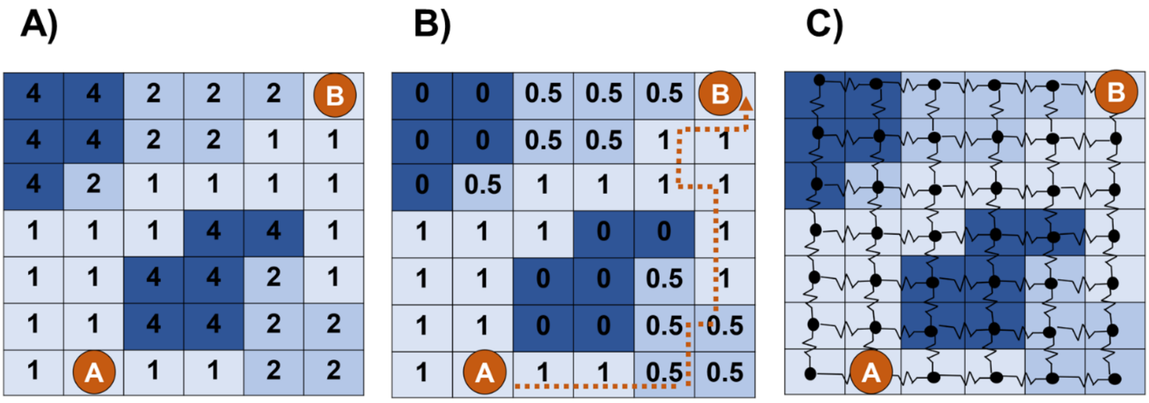

Fig 2. Schematic representation of random-walk commute time and circuit theory algorithms. A, Raster surface with each grid cell provided with a resistance value (per-cell resistance increases with darker colour and values) based on the ResistanceGA optimisation process. Orange circles are focal nodes, labelled as A and B, representing sampling locations. **B**,In the random-walk commute time algorithm, the raster grid is converted into a transition matrix with, *p*, probability that a random walker will step on that cell. Probabilities are inversely proportional to the resistance values in grid cells in **A**. Commute distance is calculated from the expected time this random walker will travel from focal node A to focal node B, back and forth, through a set of paths [16]. **C**, In circuit theory, raster grids are converted into a circuit network in which nodes (black dots) are connected by edges with resistors weighted inversely proportional to the resistance values in grid cells in **A**. If we apply a 1-amp current source to focal node (A), current will flow through all nodes connected by weighted resistors until it reaches a grounded focal node (B). Resistance distance is the accumulative passage of this current through these resistors averaged from the total available paths [17].

References.

1. Manel S, Schwartz MK, Luikart G, Taberlet P. Landscape genetics: combining landscape ecology and population genetics. Trends Ecol Evol. 2003;18: 189–197. doi:10.1016/S0169-5347(03)00008-9
2. Manel S, Holderegger R. Ten years of landscape genetics. Trends Ecol Evol. 2013;28: 614–621. doi:10.1016/j.tree.2013.05.012
3. Taylor PD, Fahrig L, Henein K, Merriam G. Connectivity is a vital element of landscape structure. NCASI Tech Bull. NCASI; 1999 May. doi:10.2307/3544927
4. STORFER A, MURPHY MA, SPEAR SF, HOLDEREGGER R, WAITS LP. Landscape genetics: where are we now? Mol Ecol. 2010;19: 3496–3514. doi:10.1111/j.1365-294X.2010.04691.x
5. LANDGUTH EL, FEDY BC, OYLER-McCANCE SJ, GAREY AL, EMEL SL, MUMMA M, et al. Effects of sample size, number of markers, and allelic richness on the detection of spatial genetic pattern. Mol Ecol Resour. 2012;12: 276–284. doi:10.1111/j.1755-0998.2011.03077.x
6. Wagner HH, Fortin MJ. A conceptual framework for the spatial analysis of landscape genetic data. Conserv Genet. 2013;14: 253–261. doi:10.1007/s10592-012-0391-5
7. Spear SF, Cushman SA, McRae BH. Resistance Surface Modeling in Landscape Genetics. Landscape Genetics. Chichester, UK: John Wiley & Sons, Ltd; 2015. pp. 129–148. doi:10.1002/9781118525258.ch08
8. Balkenhol N, Waits LP, Dezzani RJ. Statistical approaches in landscape genetics: an evaluation of methods for linking landscape and genetic data. Ecography (Cop). 2009;32: 818–830. doi:10.1111/j.1600-0587.2009.05807.x
9. Peterman WE. ResistanceGA : An R package for the optimization of resistance surfaces using genetic algorithms. Jarman S, editor. Methods Ecol Evol. 2018;9: 1638–1647. doi:10.1111/2041-210X.12984
10. Samir K, Idir B, Serra R, Brahim B, Aicha A. Genetic algorithm based objective functions comparative study for damage detection and localization in beam structures. Journal of Physics: Conference Series. Institute of Physics Publishing; 2015. doi:10.1088/1742-6596/628/1/012035
11. Sipper M, Urbanowicz RJ, Moore JH. To know the objective is not (necessarily) to know the objective function. BioData Mining. BioMed Central Ltd.; 2018. doi:10.1186/s13040-018-0182-8
12. Clarke RT, Rothery P, Raybould AF. Confidence limits for regression relationships between distance matrices: Estimating gene flow with distance. J Agric Biol Environ Stat. 2002;7: 361–372. doi:10.1198/108571102320
13. Row JR, Knick ST, Oyler-McCance SJ, Lougheed SC, Fedy BC. Developing approaches for linear mixed modeling in landscape genetics through landscape-directed dispersal simulations. Ecol Evol. 2017;7: 3751–3761. doi:10.1002/ece3.2825
14. Shirk AJ, Landguth EL, Cushman SA. A comparison of regression methods for model selection in individual-based landscape genetic analysis. Mol Ecol Resour. 2018;18: 55–67. doi:10.1111/1755-0998.12709
15. Fouss F, Pirotte A, Renders JM, Saerens M. Random-walk computation of similarities

between nodes of a graph with application to collaborative recommendation. IEEE Trans Knowl Data Eng. 2007;19: 355–369. doi:10.1109/TKDE.2007.46
16. van Etten J. R package gdistance: Distances and routes on geographical grids. J Stat Softw. 2017;76. doi:10.18637/jss.v076.i13
17. McRae BH, Dickson BG, Keitt TH, Shah VB. USING CIRCUIT THEORY TO MODEL CONNECTIVITY IN ECOLOGY, EVOLUTION, AND CONSERVATION. Ecology. 2008;89: 2712–2724. doi:10.1890/07-1861.1
18. Kivimäki I, Shimbo M, Saerens M. Developments in the theory of randomized shortest paths with a comparison of graph node distances. Phys A Stat Mech its Appl. 2014;393: 600–616. doi:10.1016/j.physa.2013.09.016
19. Shah VB, Mcrae B. Circuitscape: A Tool for Landscape Ecology. In: Varoquaux G, Vaught T, Millman J, editors. Proceedings of the 7th Python in Science Conference (SciPy 2008). 2008. pp. 62–66. Available: https://circuitscape.org/pubs/Shah_McRae_Circuitscape_Python_Scipy08.pdf
