# Supplementary material for: Population genomics and geographic dispersal in Chagas disease vectors: Landscape drivers and evidence of possible adaptation to the domestic setting
Source: PLoS Genet. 2022 Feb 4;18(2):e1010019. doi: 10.1371/journal.pgen.1010019 (PMC8849464; doi:10.1371/journal.pgen.1010019)
Supplement: S1 Fig — (PDF) [file pgen.1010019.s005.pdf]

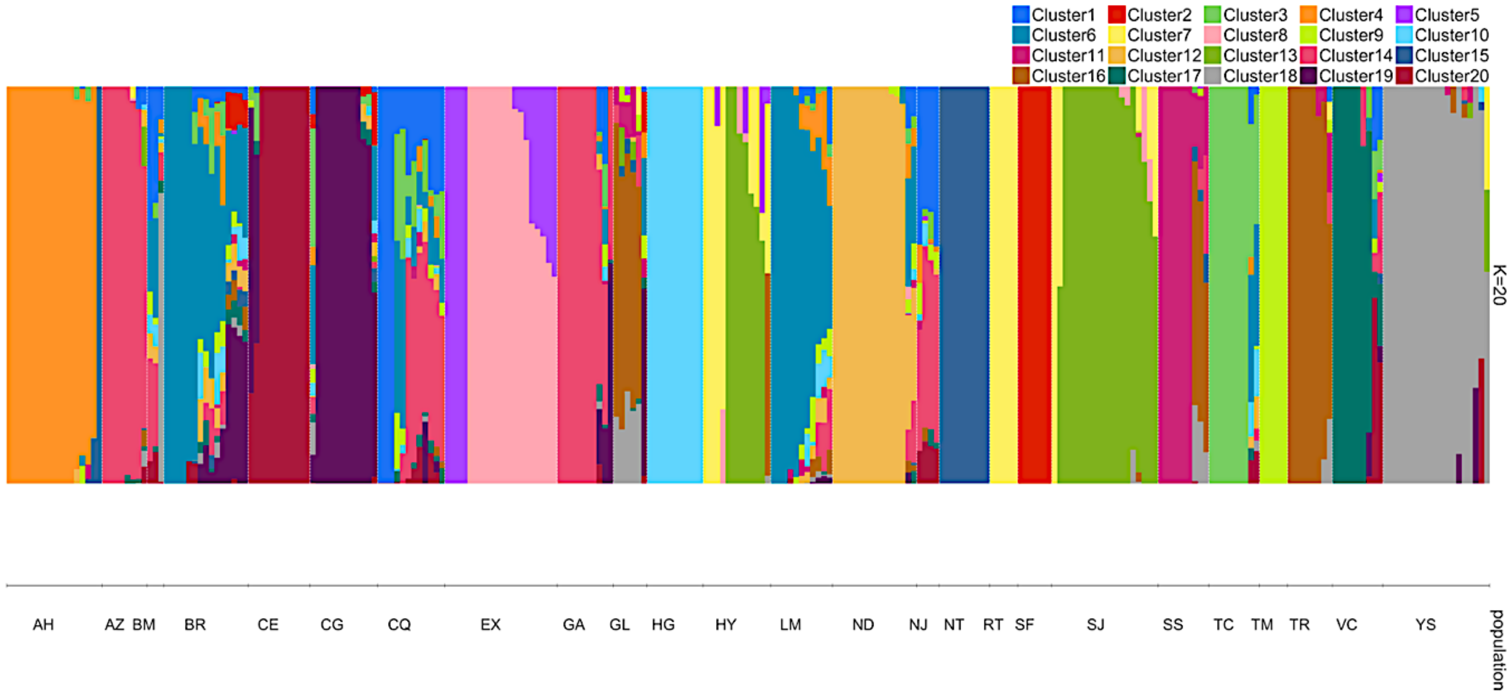

S1 Fig. Admixture bar plot of triatomine ancestries in Loja assuming *K* = 20 ancestral populations. Bars are triatomine samples divided by *K* colours proportional to the likelihood of genetic membership to a given ancestral population.
