# Supplementary material for: Population genomics and geographic dispersal in Chagas disease vectors: Landscape drivers and evidence of possible adaptation to the domestic setting
Source: PLoS Genet. 2022 Feb 4;18(2):e1010019. doi: 10.1371/journal.pgen.1010019 (PMC8849464; doi:10.1371/journal.pgen.1010019)
Supplement: S2 Fig — (PDF) [file pgen.1010019.s006.pdf]

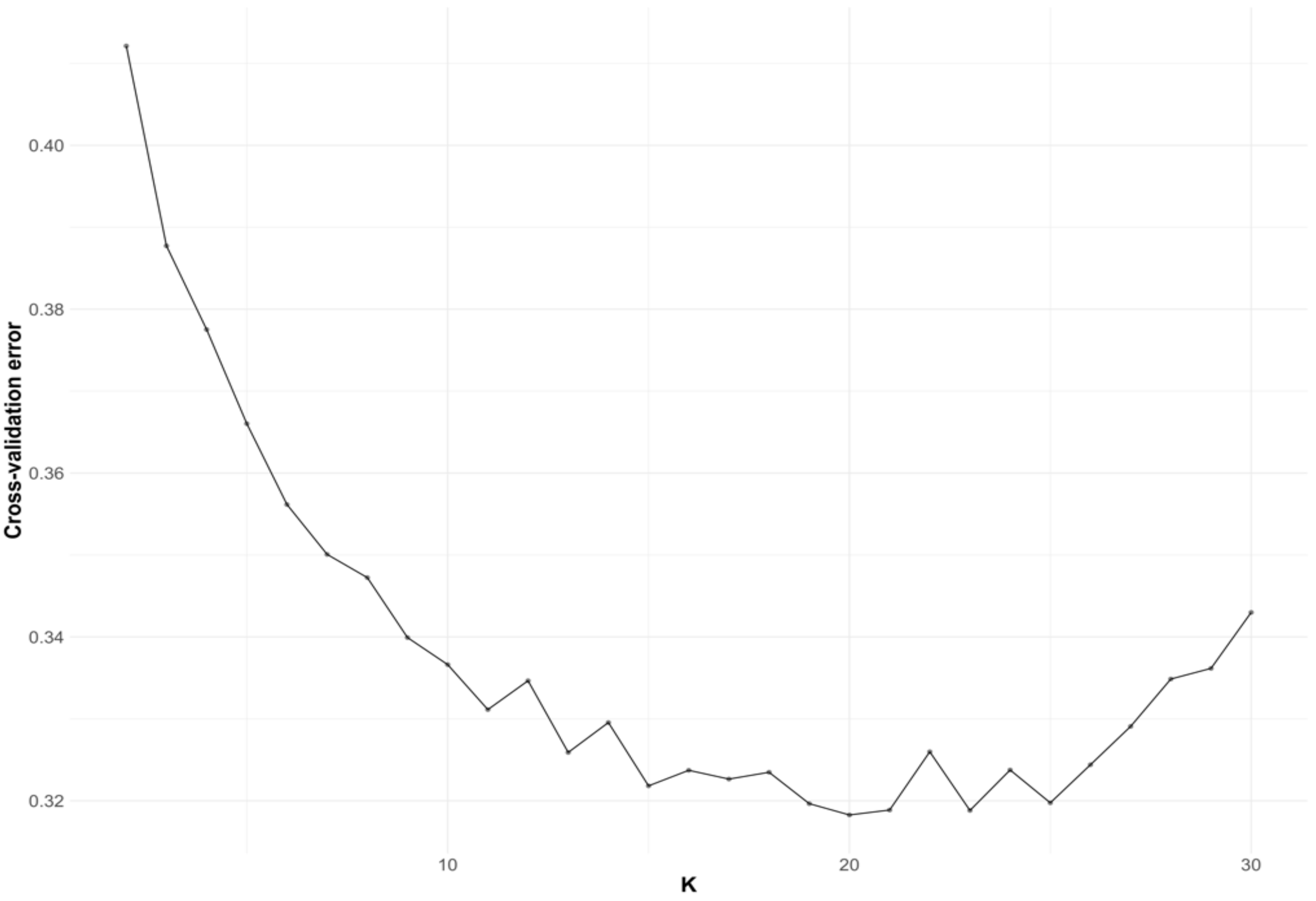

S2 Fig. Admixture analysis cross-validation error plot. Relationship between cross-validation error and *K* = 2 – 30 in Admixture runs. The optimal *K* ancestral populations is 20 as indicated by the lowest cross-validation error.
