# Supplementary material for: Population genomics and geographic dispersal in Chagas disease vectors: Landscape drivers and evidence of possible adaptation to the domestic setting
Source: PLoS Genet. 2022 Feb 4;18(2):e1010019. doi: 10.1371/journal.pgen.1010019 (PMC8849464; doi:10.1371/journal.pgen.1010019)
Supplement: S3 Fig — (PDF) [file pgen.1010019.s007.pdf]

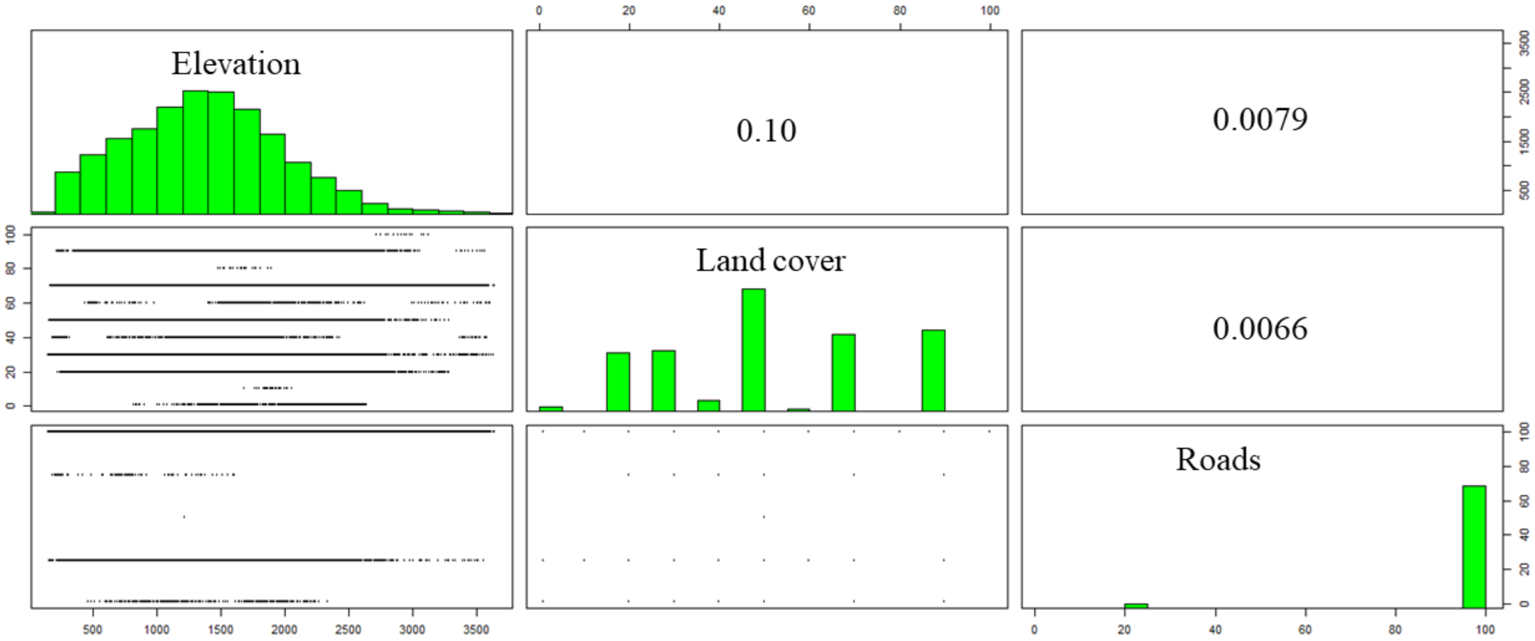

S3 Fig. Scatterplot matrix showing the relation between raster surfaces. Low correlation between relief-land cover (0.10), relief-roads (0.0079) and land cover-roads (0.0066) is observed.
