# Supplementary material for: Population genomics and geographic dispersal in Chagas disease vectors: Landscape drivers and evidence of possible adaptation to the domestic setting
Source: PLoS Genet. 2022 Feb 4;18(2):e1010019. doi: 10.1371/journal.pgen.1010019 (PMC8849464; doi:10.1371/journal.pgen.1010019)
Supplement: S4 Fig — (PDF) [file pgen.1010019.s008.pdf]

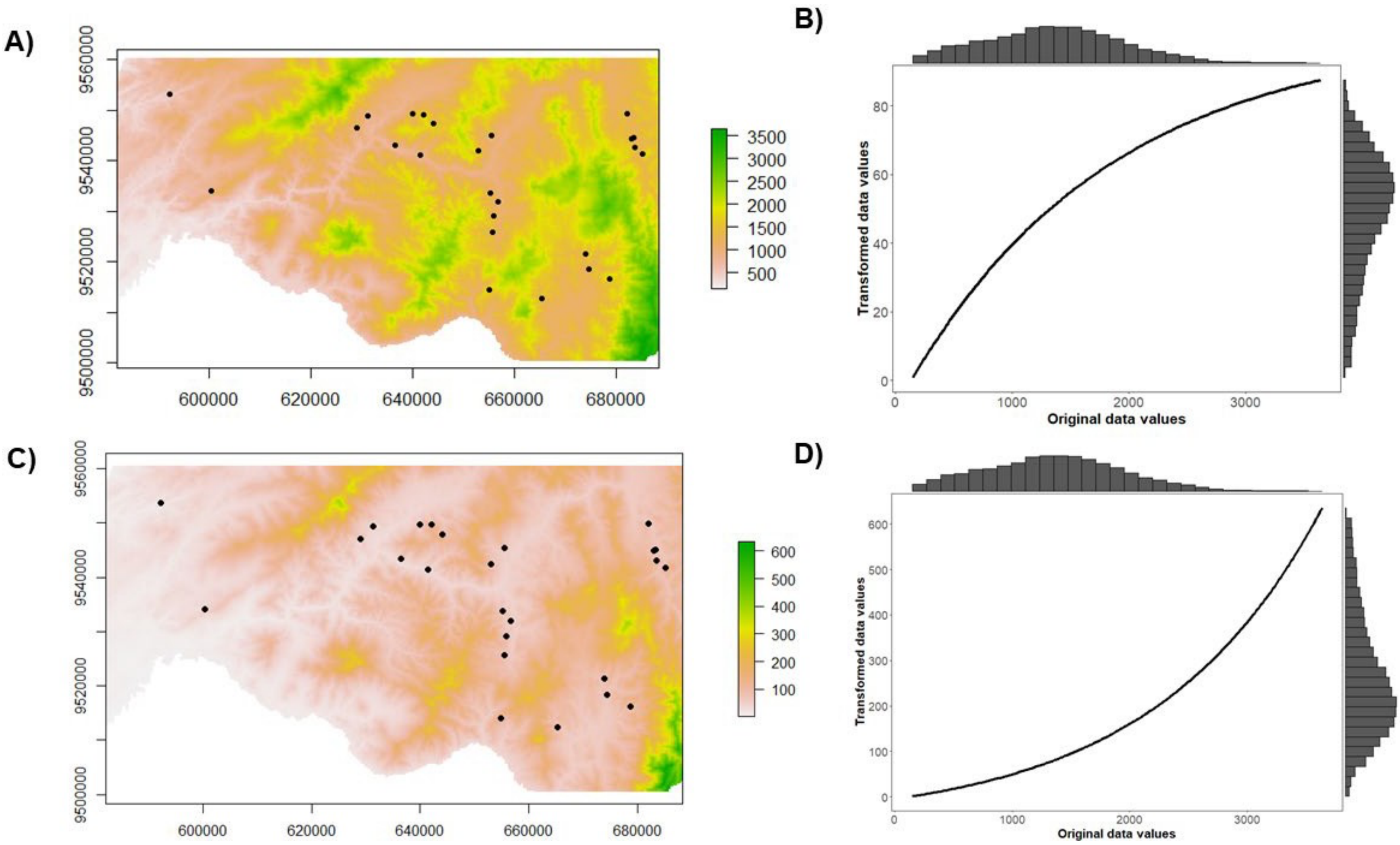

S4 Fig. Comparison between original and optimised relief resistance surfaces. Dots in **a** and **c** represent the sample location coordinates in UTM. Histograms in **b** and **d** represent the frequency of original and transformed altitude values. **a,** original altitude values of the study area. **b**, our initial hypothesis suggested an increase of cost of movement as altitude increase (monomolecular transformation) with the higher resistance values above 2,000 m.a.s.l. **c**, Resistance surface for relief showing optimised resistance values. **d**, Relief is suggested to follow a monotonical increase of cost of movement as relief increase (inverse-reverse transformation) with the highest resistance at approximately under 2,414 m.a.s.l.
