# Supplementary material for: Population genomics and geographic dispersal in Chagas disease vectors: Landscape drivers and evidence of possible adaptation to the domestic setting
Source: PLoS Genet. 2022 Feb 4;18(2):e1010019. doi: 10.1371/journal.pgen.1010019 (PMC8849464; doi:10.1371/journal.pgen.1010019)
Supplement: S5 Fig — (PDF) [file pgen.1010019.s009.pdf]

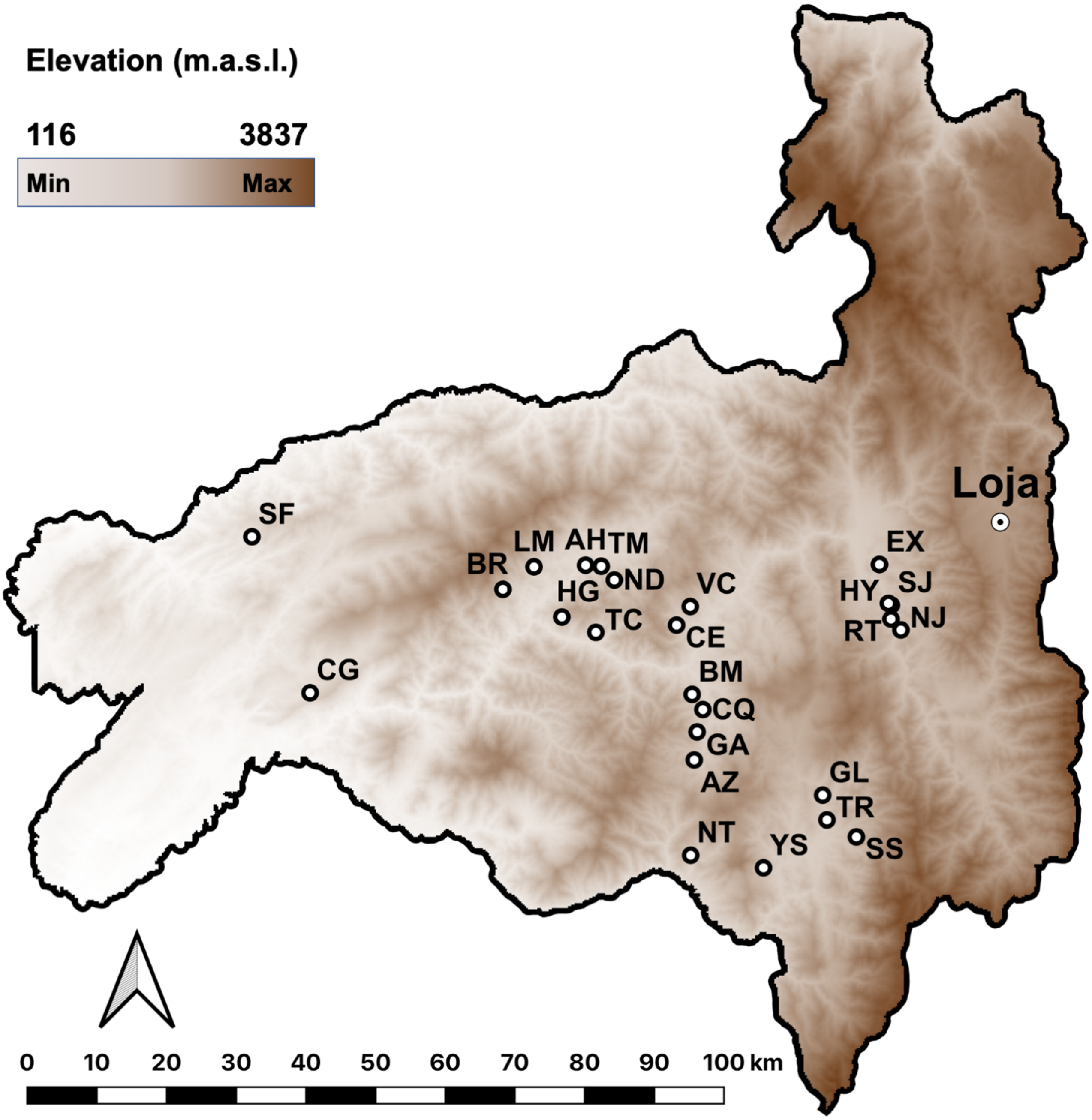


**S5 Fig. Relief of Loja, Ecuador.** Dots show the location of the 25 sampled communities at different altitudes across Loja. Relief is represented by a digital elevation model of brown colour gradient showing higher altitudes in dark shades of brown. Source map: https://www.usgs.gov/centers/eros/science/usgs-eros-archive-digital-elevation-global-multi-resolution-terrain-elevation.
