# Supplementary material for: Population genomics and geographic dispersal in Chagas disease vectors: Landscape drivers and evidence of possible adaptation to the domestic setting
Source: PLoS Genet. 2022 Feb 4;18(2):e1010019. doi: 10.1371/journal.pgen.1010019 (PMC8849464; doi:10.1371/journal.pgen.1010019)
Supplement: S6 Fig — (PDF) [file pgen.1010019.s010.pdf]

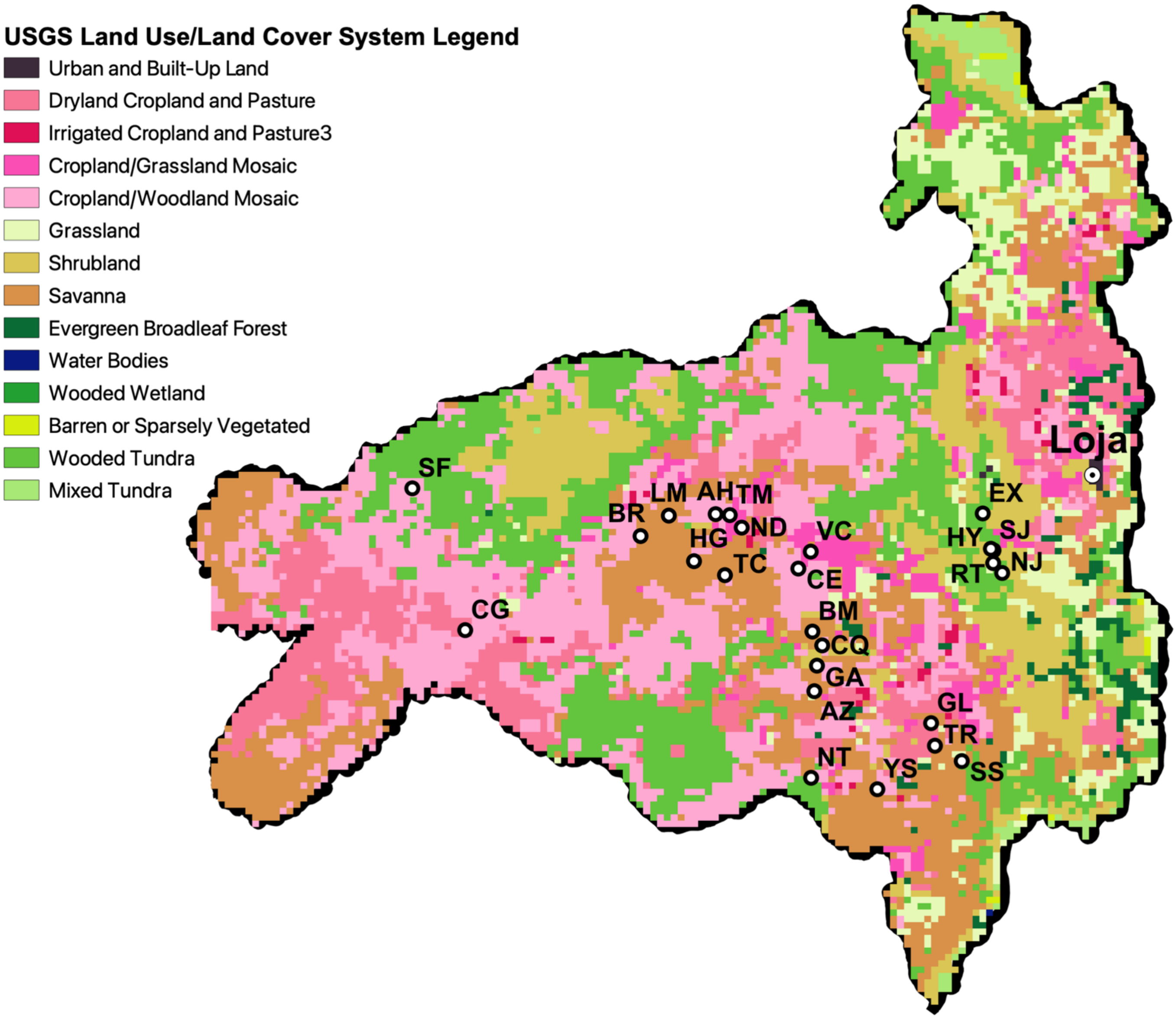

S6 Fig. Map of the land cover types present in Loja, Ecuador. Dots show the 25 communities sampled in Loja at different land cover types. Different colours indicated one of the USGS Land Use/Land Cover System Legend categories present in Loja. Source map: www.usgs.gov/media/images/south-america-land-cover-characteristics-data-base-version-20
