# Supplementary material for: Population genomics and geographic dispersal in Chagas disease vectors: Landscape drivers and evidence of possible adaptation to the domestic setting
Source: PLoS Genet. 2022 Feb 4;18(2):e1010019. doi: 10.1371/journal.pgen.1010019 (PMC8849464; doi:10.1371/journal.pgen.1010019)
Supplement: S7 Fig — (PDF) [file pgen.1010019.s011.pdf]

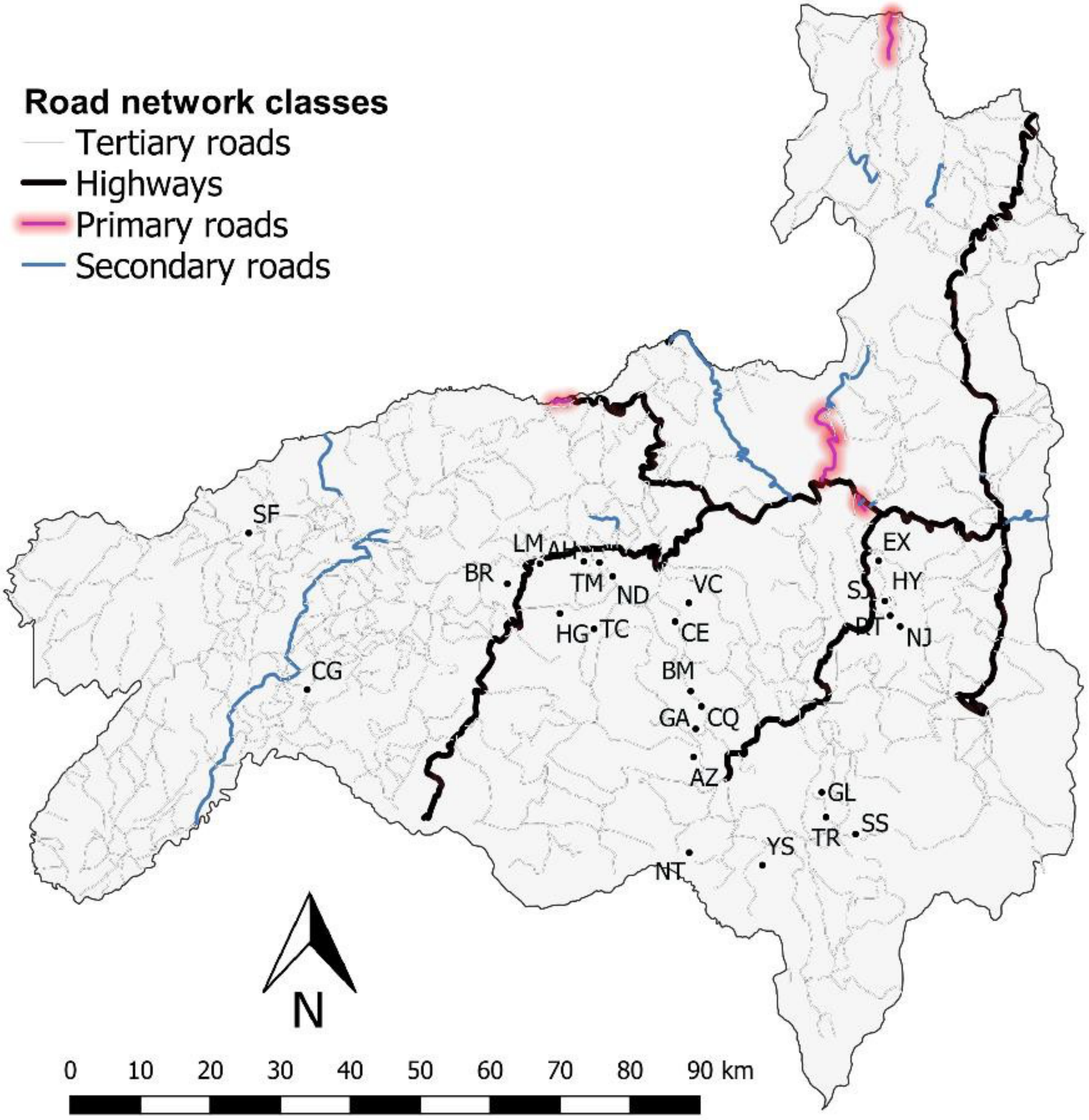

S7 Fig. Map of the road network of Loja, Ecuador. Dots show the location of the 25 sampled communities in Loja. Road categories are colour-coded to represent tertiary (grey thin line), highways (bold black line), primary (highlighted red) and secondary (light blue thin line) roads. Source map: dataportaal.pbl.nl/downloads/GRIP4/GRIP4_Region2_vector_shp.zip
