# Supplementary material for: Population genomics and geographic dispersal in Chagas disease vectors: Landscape drivers and evidence of possible adaptation to the domestic setting
Source: PLoS Genet. 2022 Feb 4;18(2):e1010019. doi: 10.1371/journal.pgen.1010019 (PMC8849464; doi:10.1371/journal.pgen.1010019)
Supplement: S8 Fig — (PDF) [file pgen.1010019.s012.pdf]

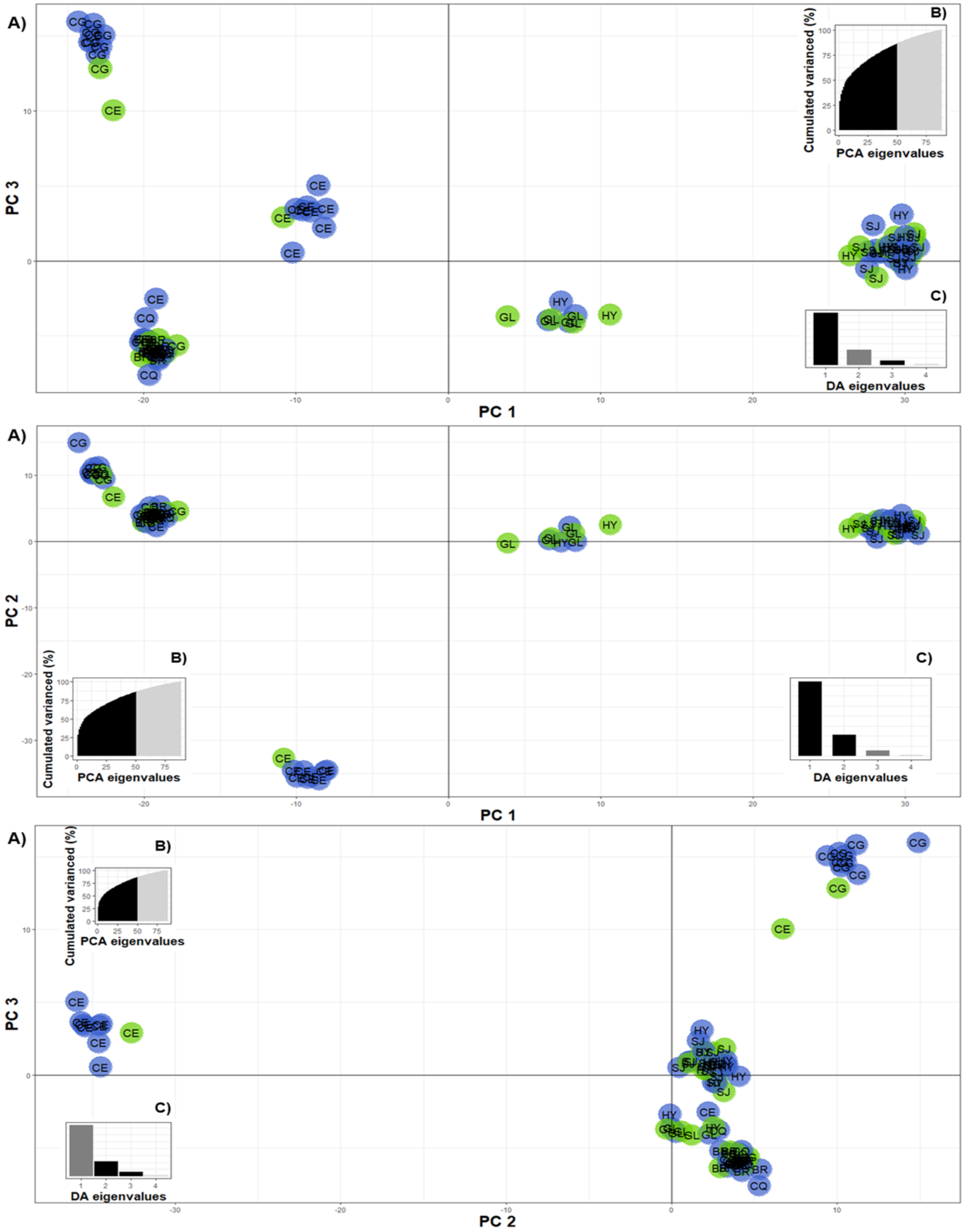

A)
B)
C)
PC 1
PC 2
PC 3
Cumulated varianced (%)
PCA eigenvalues
DA eigenvalues
CG
CE
CQ
BR
GL
HY
SJ

S8 Fig. Discriminant analysis of principal components (DAPC) scatter plots of all possible PCs axes combinations comparing ecotope vs collection site in 89 samples using 2,552 SNP markers. a, The scatter plots show different PCs combinations of the discriminant analysis eigenvalues for this small dataset. Five clusters can be seen in the scatter plots with dots representing individual samples coloured-coded to indicate their domestic (blue) or wild (green) ecotope of collection. Dots 4-digit label indicate the community of collection. Insets show **b** the percentage of cumulated variance explained (86%) by the PCs retained (black area) in the DAPC and **c** the different combinations of eigenvalues (black bars) for the discriminant functions of the discriminant analysis.
