# Supplementary material for: Population genomics and geographic dispersal in Chagas disease vectors: Landscape drivers and evidence of possible adaptation to the domestic setting
Source: PLoS Genet. 2022 Feb 4;18(2):e1010019. doi: 10.1371/journal.pgen.1010019 (PMC8849464; doi:10.1371/journal.pgen.1010019)
Supplement: S1 Table — (PDF) [file pgen.1010019.s013.pdf]

S1 Table. *Rhodnius ecuadoriensis and Rhodnius prolixus* (out group) samples proccessed in this study. Community name (collection site), 2-letter ID labels (code), province (Province), country 3-letter ISO code (Country), initial processed samples (N_0), samples included after genotyping and quality control (N_i), geographic coordinates (Latitude, Longitude), altitude, and ecotope (Domestic/Wild) of collection (Ecotope) is provided. Collection sites and samples included in this study are highlighted in grey.

Collection site	Code	Province	Country	N_0	N_i	Latitude	Longitude	Average altitude	Ecotope	
									Domestic	Wild
Mangahurquillo	MG	Loja	ECU	5	0	-4.05806	-80.26807	279.8	-	-
San Francisco	SF	Loja	ECU	10	6	-4.007935	-80.171247	857	6	-
La Cienega	CG	Loja	ECU	20	13	-4.20966	-80.09604	831.6	9	4
Algarrobillo	AB	Loja	ECU	10	0	-4.17	-80.06	766	-	-
Bramaderos	BR	Loja	ECU	20	15	-4.075825	-79.846452	1066.9	7	8
Limones	LM	Loja	ECU	12	11	-4.046738	-79.80624	1111.6	11	-
Sabanilla	SA	Loja	ECU	4	0	-4.03935	-79.758887	875	-	-
Ashimingo	AH	Loja	ECU	20	18	-4.04462	-79.7396	874	18	-
Naranjo Dulce	ND	Loja	ECU	16	15	-4.06381	-79.70243	1212.8	15	-
Higida	HG	Loja	ECU	10	10	-4.111565	-79.770365	1328	10	-
Tacoranga	TC	Loja	ECU	15	10	-4.13144	-79.726345	1182.1	10	-
Vega del Carmen	VC	Loja	ECU	19	11	-4.09797	-79.60424	1378	11	-
Coamine	CE	Loja	ECU	22	11	-4.122058796	-79.6219098	1360.6	9	2
Chirimoyos	CY	Loja	ECU	3	0	-4.128295351	-79.54458683	1167	-	-
Bella Maria	BM	Loja	ECU	19	3	-4.211679	-79.601831	1109	3	-
Chaquizhca	CQ	Loja	ECU	24	11	-4.231328	-79.587898	1189.5	9	2
Guara	GA	Loja	ECU	19	10	-4.26021	-79.5954	1306	10	-
Jacapo de Quillanga	JQ	Loja	ECU	2	0	-4.36387	-79.41602	1542	-	-
La Extensa	EX	Loja	ECU	23	20	-4.043671	-79.359601	1250.8	20	-
San Jacinto	SJ	Loja	ECU	22	19	-4.09617	-79.34427	1241.5	10	9
El Huayco	HY	Loja	ECU	73	13	-4.093951	-79.348045	1278	10	3
Camayos	YS	Loja	ECU	20	19	-4.435818	-79.509447	1328	19	-
Quisanga	QS	Loja	ECU	2	0	-4.42387	-79.51677	1640	-	-
Galapagos	GL	Loja	ECU	20	7	-4.341976	-79.432994	1315.1	3	4
Tuburo	TR	Loja	ECU	19	8	-4.37399	-79.42748	1236	8	-
Santa Rosa	SS	Loja	ECU	19	10	-4.3958	-79.38939	1542.9	10	-
Naranjillo	NJ	Loja	ECU	10	4	-4.12836	-79.3319	1261	4	-
Ardanza	AZ	Loja	ECU	8	8	-4.29646	-79.59912	1384.6	-	8
San Antoni de Taparuca	NT	Loja	ECU	10	9	-4.41953	-79.60369	1188.17	-	9
Santa Rita	RT	Loja	ECU	10	6	-4.11422	-79.34474	1256.8	-	6
Tamarindo	TM	Loja	ECU	10	5	-4.046004	-79.719427	1027	5	-
Rhodnius Prolixus	RP	Lara	VEN	6	6	Unknown	Unknown	-	6	-
