# Supplementary material for: Population genomics and geographic dispersal in Chagas disease vectors: Landscape drivers and evidence of possible adaptation to the domestic setting
Source: PLoS Genet. 2022 Feb 4;18(2):e1010019. doi: 10.1371/journal.pgen.1010019 (PMC8849464; doi:10.1371/journal.pgen.1010019)
Supplement: S2 Table — (PDF) [file pgen.1010019.s014.pdf]

S2 Table. *Rhodnius ecuadoriensis* population genetic summary statistics. Community name (Collection site), 2-letter ID labels (Code), sample collection ecotope (ecotope), number of samples (n), mean observed heterozygosity (HO), mean gene diversity (H_E), inbreeding index (F_{IS}) and allelic richness (A_r) is provided. Bold H_O, H_E, F_{IS} and A_r values inside yellow boxes indicated significant differences existed between domestic and wild mean diversity according to randomisation and t-test permutations.

Collection site	***Code***	***ecotope***	***n***	H_O	H_E	F_{IS}	A_r
San Francisco	SF	domestic	6	0.21	0.17	-0.19	1.35
La Cienega	CG	domestic	9	**0.21**	**0.21**	**0.01**	1.36
		wild	4	**0.23**	**0.22**	**-0.05**	1.38
Bramaderos	BR	domestic	7	**0.16**	**0.17**	**0.10**	**1.30**
		wild	8	**0.23**	**0.22**	**-0.03**	**1.39**
Limones	LM	domestic	11	0.20	0.21	0.04	1.44
Ashimingo	AH	domestic	18	0.20	0.19	0.01	1.40
Naranjo Dulce	ND	domestic	15	0.19	0.17	-0.04	1.37
Higida	HG	domestic	10	0.17	0.16	-0.02	1.35
Tacoranga	TC	domestic	10	0.16	0.18	0.11	1.37
Vega del Carmen	VC	domestic	11	0.15	0.16	0.10	1.34
Coamine	CE	domestic	9	**0.16**	**0.16**	**0.05**	**1.28**
		wild	2	**0.19**	**0.21**	**-0.06**	**1.31**
Bella Maria	BM	domestic	3	0.22	0.21	-0.08	1.41
Chaquizhca	CQ	domestic	9	0.18	0.19	**0.08**	**1.34**
		wild	2	0.17	0.18	**-0.09**	**1.27**
Guara	GA	domestic	10	0.22	0.19	-0.08	1.41
La Extensa	EX	domestic	20	0.11	0.09	-0.09	1.19

San Jacinto	SJ	domestic	10	0.14	0.12	-0.12	1.21
		wild	9	0.14	0.12	-0.13	1.21
El Huayco	HY	domestic	10	**0.12**	**0.12**	**-0.02**	**1.21**
		wild	3	**0.17**	**0.15**	**-0.17**	**1.25**
Camayos	YS	domestic	19	0.19	0.16	-0.08	1.34
Galapagos	GL	domestic	3	**0.16**	**0.16**	**-0.08**	**1.26**
		wild	4	**0.20**	**0.21**	**0.022**	**1.35**
Tuburo	TR	domestic	8	0.17	0.16	-0.03	1.34
Santa Rosa	SS	domestic	10	0.14	0.15	0.09	1.32
Naranjillo	NJ	domestic	4	0.19	0.19	-0.03	1.39
Ardanza	AZ	wild	8	0.17	0.18	0.03	1.37
San Antonio de Taparuca	NT	wild	9	0.15	0.12	-0.24	1.25
Santa Rita	RT	wild	6	0.13	0.11	-0.13	1.23
Tamarindo	TM	domestic	5	0.15	0.14	-0.03	1.29
