# Supplementary material for: Population genomics and geographic dispersal in Chagas disease vectors: Landscape drivers and evidence of possible adaptation to the domestic setting
Source: PLoS Genet. 2022 Feb 4;18(2):e1010019. doi: 10.1371/journal.pgen.1010019 (PMC8849464; doi:10.1371/journal.pgen.1010019)
Supplement: S3 Table — (PDF) [file pgen.1010019.s015.pdf]

S3 Table. Summarised results of hierarchical differentiation of molecular variance components in 2552 SNP loci on the complete (n = 272 samples) and a small (n = 89 samples) datasets. Variance components, F-statistic (= G-statistic [1]), confidence interval (C.I. at 2.5% and 97.5%) and P-values (significance level based in randomised 999 permutation of individuals level-defined) are provided.

Source of variation	**dataset (n = 272)**				**dataset (n = 89)**			
	Variance component	F-statistic	C.I.	p-value	Variance components	F-statistic	C.I,	p-value
Among communities	151.21	0.26	0.25 0.27	0.001	149.91	0.25	0.24 0.26	0.001
Among populations within communities	-18.70	-0.04	-0.05 -0.04	0.001	-2.04	-0.004	-0.01 0.004	0.001
Among collection year within communities	52.19	0.07	0.07 0.08	0.001	30.06	0.06	0.058 0.067	0.001
Individuals within communities	-34.19	-0.001	-0.02 0.02		-18.13	0.02	-0.001 0.04	
Error	436.63				436.01			

References.

1. GOUDET J. hierfstat, a package for r to compute and test hierarchical F-statistics. Mol Ecol Notes. 2005;5: 184–186. doi:10.1111/j.1471-8286.2004.00828.x
