# Supplementary material for: Population genomics and geographic dispersal in Chagas disease vectors: Landscape drivers and evidence of possible adaptation to the domestic setting
Source: PLoS Genet. 2022 Feb 4;18(2):e1010019. doi: 10.1371/journal.pgen.1010019 (PMC8849464; doi:10.1371/journal.pgen.1010019)
Supplement: S4 Table — (PDF) [file pgen.1010019.s016.pdf]

S4 Table. GLS-MLPE model results. Estimated regression parameters with 95% confidence intervals (Lower/Upper), standard errors, t-values and p-values for the GLS-MLPE model presented in eqn 1.

	Estimate	Lower	Upper	Std. error	t value	P-value
**Intercept**	0.12	0.08	0.16	0.021	5.67	< 0.001
**Geographic distance**	0.0016	0.0014	0.0018	0.0001	14.13	< 0.001
**Ecotope Wild**	-0.07	-0.15	0.06	0.04	-1.88	0.06
**Geographic distance: Ecotope Wild**	0.0018	0.0011	0.0024	0.003	5.72	< 0.001
