# Supplementary material for: Population genomics and geographic dispersal in Chagas disease vectors: Landscape drivers and evidence of possible adaptation to the domestic setting
Source: PLoS Genet. 2022 Feb 4;18(2):e1010019. doi: 10.1371/journal.pgen.1010019 (PMC8849464; doi:10.1371/journal.pgen.1010019)
Supplement: S5 Table — (PDF) [file pgen.1010019.s017.pdf]

S5 Table. Matirx of pairwise G_{ST} values for the 25 collection sites in Loja.

	SF	CG	BR	LM	AH	TC	ND	HG	TM	VC	CE	BM	CQ	GA	AZ	NT	YS	TR	SS	GL	NJ	RT	HY	SJ	EX
**SF**	**0.00**																								
**CG**	0.29	**0.00**																							
**BR**	0.29	0.14	**0.00**																						
**LM**	0.31	0.16	0.08	**0.00**																					
**AH**	0.38	0.23	0.15	0.14	**0.00**																				
**TC**	0.40	0.25	0.17	0.17	0.24	**0.00**																			
**ND**	0.41	0.26	0.17	0.17	0.23	0.25	**0.00**																		
**HG**	0.41	0.27	0.19	0.20	0.27	0.28	0.30	**0.00**																	
**TM**	0.46	0.31	0.24	0.23	0.30	0.32	0.32	0.37	**0.00**																
**VC**	0.45	0.33	0.25	0.26	0.31	0.33	0.35	0.36	0.40	**0.00**															
**CE**	0.44	0.30	0.23	0.24	0.30	0.30	0.34	0.35	0.39	0.28	**0.00**														
**BM**	0.35	0.19	0.09	0.09	0.18	0.19	0.20	0.23	0.26	0.25	0.23	**0.00**													
**CQ**	0.35	0.21	0.13	0.13	0.19	0.19	0.22	0.25	0.28	0.24	0.24	0.08	**0.00**												
**GA**	0.37	0.22	0.17	0.17	0.24	0.26	0.26	0.30	0.33	0.28	0.27	0.15	0.15	**0.00**											
**AZ**	0.43	0.28	0.20	0.20	0.27	0.29	0.28	0.32	0.35	0.28	0.29	0.17	0.18	0.18	**0.00**										
**NT**	0.55	0.42	0.37	0.38	0.42	0.45	0.44	0.48	0.52	0.47	0.46	0.38	0.39	0.41	0.42	**0.00**									
**YS**	0.46	0.33	0.26	0.26	0.33	0.34	0.35	0.38	0.41	0.37	0.35	0.20	0.28	0.30	0.30	0.40	**0.00**								
**TR**	0.46	0.32	0.27	0.26	0.33	0.36	0.35	0.37	0.42	0.39	0.36	0.24	0.28	0.31	0.32	0.42	0.25	**0.00**							
**SS**	0.48	0.35	0.30	0.29	0.36	0.38	0.37	0.40	0.44	0.41	0.41	0.28	0.31	0.35	0.35	0.44	0.29	0.22	**0.00**						
**GL**	0.37	0.22	0.17	0.17	0.24	0.26	0.26	0.30	0.32	0.29	0.28	0.14	0.18	0.22	0.23	0.35	0.18	0.13	0.19	**0.00**					
**NJ**	0.39	0.23	0.15	0.15	0.21	0.22	0.24	0.28	0.30	0.27	0.24	0.11	0.11	0.17	0.18	0.41	0.29	0.31	0.33	0.20	**0.00**				
**RT**	0.62	0.52	0.46	0.44	0.49	0.52	0.51	0.55	0.57	0.50	0.51	0.44	0.45	0.48	0.46	0.55	0.46	0.46	0.49	0.40	0.46	**0.00**			
**HY**	0.59	0.48	0.42	0.41	0.45	0.48	0.47	0.51	0.53	0.46	0.47	0.39	0.41	0.43	0.41	0.52	0.42	0.41	0.44	0.36	0.42	0.07	**0.00**		
**SJ**	0.61	0.51	0.45	0.44	0.48	0.51	0.50	0.54	0.55	0.49	0.50	0.42	0.44	0.46	0.44	0.54	0.45	0.44	0.48	0.39	0.45	0.08	0.02	**0.00**	
**EX**	0.65	0.55	0.50	0.49	0.53	0.56	0.55	0.59	0.60	0.55	0.55	0.48	0.49	0.51	0.49	0.59	0.50	0.51	0.54	0.45	0.50	0.19	0.12	0.15	**0.00**
