# Supplementary material for: Population genomics and geographic dispersal in Chagas disease vectors: Landscape drivers and evidence of possible adaptation to the domestic setting
Source: PLoS Genet. 2022 Feb 4;18(2):e1010019. doi: 10.1371/journal.pgen.1010019 (PMC8849464; doi:10.1371/journal.pgen.1010019)
Supplement: S6 Table — (PDF) [file pgen.1010019.s018.pdf]

**S6 Table. Spearman correlation test, rho, between raster surfaces previous to optimization with ResistanceGA.**

| Surface 1 | Surface 2 | Spearman's rho |
|---|---|---|
| Relief | Land cover | -0.1315174 |
| Relief | Roads | 0.01405202 |
| Roads | Land cover | 0.03044303 |
