# Supplementary material for: Population genomics and geographic dispersal in Chagas disease vectors: Landscape drivers and evidence of possible adaptation to the domestic setting
Source: PLoS Genet. 2022 Feb 4;18(2):e1010019. doi: 10.1371/journal.pgen.1010019 (PMC8849464; doi:10.1371/journal.pgen.1010019)
Supplement: S7 Table — (PDF) [file pgen.1010019.s019.pdf]

S7 Table. Global Land Cover 2000 project Legend. Aggregated from regional classes using United Nations Food and Agricultural Organisation (FAO) Land Cover Classification System (LCCS). DIVA-GIS programme at https://www.diva-gis.org/.

	**GLC Global Class (according to LCCS terminology)**
1	**Tree Cover, broadleaved, evergreen**<br>*LCCS >15% tree cover, tree height >3m*<br>(Examples of sub-classes at regional level* :<br>*closed > 40% tree cove; open 15-40% tree cover)*
2	**Tree Cover, broadleaved, deciduous, closed**
3	**Tree Cover, broadleaved, deciduous, open**<br>*(open 15-40% tree cover)*
4	**Tree Cover, needle-leaved, evergreen**
5	**Tree Cover, needle-leaved, deciduous**
6	**Tree Cover, mixed leaf type**
7	**Tree Cover, regularly flooded, fresh water** (& brackish)
8	**Tree Cover, regularly flooded, saline water,**<br>**(**daily variation of water level)
9	**Mosaic:**<br>**Tree cover / Other natural vegetation**
10	**Tree Cover, burnt**
11	**Shrub Cover, closed-open, evergreen**<br>(Examples of sub-classes at reg. level *: (i) sparse tree layer)
12	**Shrub Cover, closed-open, deciduous**<br>(Examples of sub-classes at reg. level *: (i) sparse tree layer)
13	**Herbaceous Cover, closed-open**<br>(Examples of sub-classes at regional level *:<br>(i) natural, (ii) pasture, (iii) sparse trees or shrubs)
14	**Sparse Herbaceous or sparse Shrub Cover**

15	**Regularly flooded Shrub and/or Herbaceous Cover**
16	**Cultivated and managed areas**<br>(Examples of sub-classes at reg. level *:<br>(i) terrestrial; (ii) aquatic (=flooded during cultivation), and under terrestrial: (iii) tree crop & shrubs (perennial), (iv) herbaceous crops (annual), non-irrigated, (v) herbaceous crops (annual), irrigated)
17	**Mosaic:**<br>**Cropland / Tree Cover / Other natural vegetation**
18	**Mosaic:**<br>**Cropland / Shrub or Grass Cover**
19	**Bare Areas**
20	**Water Bodies** (natural & artificial)
21	**Snow and Ice** (natural & artificial)
22	**Artificial surfaces and associated areas**
