# Supplementary material for: Population genomics and geographic dispersal in Chagas disease vectors: Landscape drivers and evidence of possible adaptation to the domestic setting
Source: PLoS Genet. 2022 Feb 4;18(2):e1010019. doi: 10.1371/journal.pgen.1010019 (PMC8849464; doi:10.1371/journal.pgen.1010019)
Supplement: S8 Table — (PDF) [file pgen.1010019.s020.pdf]

S8 Table. Land cover reclassified values. Original and reclassified resistance values for the different land cover categories are provided.

GLC GLOBAL CLASS (ACCORDING TO LCCS TERMINOLOGY)	ORIGINAL VALUE	TRANSFORMED VALUE
TREE COVER, BROADLEAVED, EVERGREEN	1	70
TREE COVER, BROADLEAVED, DECIDUOUS, CLOSED	2	60
TREE COVER, REGULARLY FLOODED, FRESH WATER (& BRACKISH)	7	90
SHRUB COVER, CLOSED-OPEN, DECIDUOUS	12	30
HERBACEOUS COVER, CLOSED-OPEN	13	40
SPARSE HERBACEOUS OR SPARSE SHRUB COVER	14	50
REGULARLY FLOODED SHRUB AND/OR HERBACEOUS COVER	15	80
CULTIVATED AND MANAGED AREAS	16	1
MOSAIC: CROPLAND / TREE COVER / OTHER NATURAL VEGETATION	17	20
MOSAIC: CROPLAND / SHRUB OR GRASS COVER	18	10
WATER BODIES (NATURAL & ARTIFICIAL)	20	100
