# Supplementary material for: Population genomics and geographic dispersal in Chagas disease vectors: Landscape drivers and evidence of possible adaptation to the domestic setting
Source: PLoS Genet. 2022 Feb 4;18(2):e1010019. doi: 10.1371/journal.pgen.1010019 (PMC8849464; doi:10.1371/journal.pgen.1010019)
Supplement: S9 Table — (PDF) [file pgen.1010019.s021.pdf]

S9 Table. Roads reclassified values. Original and reclassified resistance values for the different road classes are provided.

**TERTIARY ROADS**	0	25
**HIGHWAYS**	1	1
**PRIMARY ROADS**	2	50
**SECONDARY ROADS**	3	75
**NO ROADS**	4	100
