# Supplementary material for: Population genomics and geographic dispersal in Chagas disease vectors: Landscape drivers and evidence of possible adaptation to the domestic setting
Source: PLoS Genet. 2022 Feb 4;18(2):e1010019. doi: 10.1371/journal.pgen.1010019 (PMC8849464; doi:10.1371/journal.pgen.1010019)
Supplement: S10 Table — (PDF) [file pgen.1010019.s022.pdf]

**S10 Table. Original GIS data, description and collection source.**

| Original GIS data | Original format | Source |
|---|---|---|
| Relief was obtained from a digital elevation model | • Raster format.<br>• Resolution of 90 m at the equator.<br>• Minimum altitude of 105 and maximum of 3866 m.a.s.l. | Shuttle Radar Topography Mission (SRTM) database [1] (srtm.csi.cgiar.org/wp-content/uploads/files/srtm_5x5/TIFF/srtm_21_13.zip) |
| Land cover | • Raster format<br>• Resolution approximately 1 Km.<br>• Eleven distinct land cover classifications.<br>• Classification is based on the Global Land Cover (GLC) 2000 project which follows United Nations Food and Agricultural Organisation (FAO) Land Cover Classification System (LCCS). Table 7 | DIVA-GIS programme from GLC2000 [2]<br><br>(biogeo.ucdavis.edu/data/diva/cov/ECU_cov.zip) |
| Road network | • Vector format.<br>• Five road categories: highways, primary, secondary, tertiary and no roads. | Global Roads Inventory Project (GRIP) dataset [3] (dataportaal.pbl.nl/downloads/GRIP4/GRIP4_Region2_vector_shp.zip). |

## References


1. Jarvis A, Reuter HI, Nelson A, Guevara E. Hole-filled seamless SRTM data V4. In:

International Centre for Tropical Agriculture (CIAT) [Internet]. Available: https://srtm.csi.cgiar.org

2. European Commission. Global Land Cover 2000 database. In: Joint Research Centre. 2003.

3. Meijer JR, Huijbregts MAJ, Schotten KCGJ, Schipper AM. Global patterns of current and future road infrastructure. Environ Res Lett. 2018;13. doi:10.1088/1748-9326/aabd42
