# Supplementary material for: Population genomics and geographic dispersal in Chagas disease vectors: Landscape drivers and evidence of possible adaptation to the domestic setting
Source: PLoS Genet. 2022 Feb 4;18(2):e1010019. doi: 10.1371/journal.pgen.1010019 (PMC8849464; doi:10.1371/journal.pgen.1010019)
Supplement: Attachment — Submitted filename: Response_to_Reviewers.docx [file pgen.1010019.s023.docx]

University of Edinburgh,

The Roslin Institute,

Easter Bush Campus,

Midlothian,

EH25 9RG

Scotland

6^th^ December 2021

**Re: Revision of PLOS Genetics PGENETICS-D-21-00937**

To whom it may concern,

We would like to thank editors and reviewers for extensive and constructive comments to our manuscript. After a careful revision, my co-authors and I have amended the manuscript accordingly and a point-by-point discussion of the changes that have been made in the pages below. The comments received by the reviewers are reproduced in full (in black font) followed by our responses (in blue bold font).

We greatly appreciated the time taken in considering our manuscript and would be available to address any further questions should they arise.

Yours faithfully,

Luis Enrique Hernandez Castro

Mobile: +447452992678

Research Fellow – Centre for Tropical Livestock Genetics and Health.

University of Edinburgh

**Reviewer's Responses to Questions**

**Comments to the Authors:
Please note here if the review is uploaded as an attachment.**

**Reviewer #1:** In the manuscript, the authors used a landscape genomics approach to assay gene flow, local adaptation and dispersal of wild and domestic Rhodnius ecuadoriensis species, at 25 sites in the Loja Valley in Southern Ecuador. Previously, the same authors identified R. ecuadoriensis, which is one of the main vectors of Chagas disease, in sylvatic highlands of the same region (Grijalva et al., 2009) and showed that ecological factors such as nest type, height above sea level and distance to houses are important for the widespread distribution of sylvatic R. ecuadoriensis populations in southern Ecuador (Grijalva et al., 2012). The authors also hypothesized that the arrival of sylvatic populations to dwellings may be an important re-infestation source, which could hamper vector control efforts.
With the availability of Next-generation sequencing technologies, the authors have been taking the next steps in their analysis to study the genetic diversity in those sampled populations. They were able to recover 2,552 SNP markers across 272 R. ecuadoriensis samples collected across ecological gradients in Loja and found high levels of gene flow between multiple domestic and wild R. ecuadoriensis populations. They then screened for SNP RADseq markers under a strong signal of selection (outlier loci) using 4 different approaches: the Random Forest (RF) algorithm, redundancy analysis (RDA) constraint ordination and OutFlank and fsthet FST-outlier methods. 43 SNPs were identified with the highest classification accuracy in RF analysis. Four of those SNPs showed a strong signal of adaptation by RDA analysis. One SNP (ID 15732) flagged in all four analyses. In the absence of an available R. ecuadoriensis genome, the resulting SNPs found in the association analyses were then Blast searched against the R. prolixus annotated genome. Three SNPs were mapped to genes involved with embryogenesis and saliva production. Unfortunately, Locus ID 15732 did not give any suitable match.

This study identified some of the genomic signatures of domestication of a triatomine species which could be useful to provide a better understanding of key mechanisms of domestication and potential targets for novel vector control approaches (e.g. genetic technologies). A lot of effort has gone into the data analysis and the landscape genomic model which showed that R. ecuadoriensis dispersal is fundamentally restricted by landscape elevation and identified highly connected and isolated triatomine populations could be very useful to predict reinfestation of houses by wild R. ecuadoriensis following vector control interventions.

I recommend publication of this study in PLoS Genetics if some of the points below are addressed since I believe it will be widely appreciated by the Chagas disease research community as well as population geneticists and modelling community to adapt this thoughtful analysis to their specific research needs.

Here are some general comments:

1) There are already numerous publications that have looked at the importance of environmental conditions such as altitude, climate, vegetation types and land uses for the Triatome distribution.
In my opinion this information is important and should be presented in the study.
For example, Ramsey et al (2000) showed that differences in altitude and mean annual precipitation were important in the habitat partitioning of 8 Triatoma species and R. prolixus in the state of Oaxaca, Mexico. Another study looked at reinfestation processes in Argentina and already highlights the importance of altitude and suggests that Chagas disease control program should consider potential sources of triatomines up to 1,500 m around the dwelling to reduce adult invasion (Cecere et al., 2007). Further, Bustamante et al. (2007) published a study on environmental determinants of the distribution of Chagas disease vectors in south-eastern Guatemala.
I would therefore like to encourage the authors to expand in the introduction or discussion on what is already known about factors that affect Triatome dispersal and to highlight their unique and valuable map that shows connectivity between sites.

**We thank reviewer for advice on studies that investigated environmental conditions for triatomine distribution and suitable habitats based on presence data surveys and using regression models. We have included these references in the introduction and agree it is valuable for the reader to know methods and findings related to triatomine distribution. In addition, we also highlighted that most studies show species distribution and predicted suitability in a broad spatial context, and they do not account for probability of dispersal based on landscape functional connectivity which is the uniqueness and value of our map. We included a paragraph with reviewer suggestions in Lines 138 - 140.**

Further, it would be useful for the reader to know why those parameters (Road, Land cover and elevation) were chosen for the model over others such as average minimum temperature, rainfall/relative humidity?

**We provided information of GIS data collection and preparation in the methods section (paragraph in Lines 771 - 796), but we have expanded on why these variables were selected over other variables. Our hypothesis on landscape variables driving this triatomine specie distribution was based on previous studies on *R. ecuadoriensis* distribution (Grijalva 2014). As the reviewer suggested correctly, elevation is an important variable for many triatomines, especially those distributed in the Andean mountains range such as *R. ecuadoriensis* in Loja. Finally, roads were used to test human-driven dispersal. To avoid collinearity between variables (e.g., elevation vs temperature and precipitation) and overfitting in landscape mixed models and GA algorithms parametrisation, we narrow the analysis to our hypothesised variables. We have provided further information for the reader on variables selection in Lines 773 – 775.**

As mentioned above,

2) The authors mentioned that the current vector control strategies are insufficient and merely reactive. However, they did not provide more details in the introduction or discussion on what the current control measures are, nor how/ whether they are applied in the sampled regions. Could this be another parameter to consider?

**We have provided details and references (Lines 491 - 495) on the historical and current vector control in Loja, which has only formally focused on insecticide spraying. However, it is sporadic, limited, and variable due to resources availability. They focused on a single administrative unit for a given intervention and records on sampled regions are scarce. In landscape mixed models generally including vector control interventions may be useful, especially for populations under strong continuous insecticide spraying or other interventions. However, this is not the case for *R. ecuadoriensis* in Loja. Typically, parameters/variables in this type of models are included as grids with values spanning the study area. Unfortunately, scarce and unreliable historical vector control records in the area make difficult to consider parameters of this type.**

3) Did the collection happen throughout the year or only during a certain season? Does the abundance/composition of wild vs. domestic R. ecuadoriensis species vary across the year? How would the authors expect this to affect the model?

**We would not expect *R. ecuadoriensis* abundance have an effect in our models. Although it is correct triatomine abundance varies seasonality presence in house/wild is constant throughout the year, Moreover, this has little impact in inferring gene flow and population structure with the reliable and high-resolution markers we obtained in this population. Sampling was carried out during different surveys at different times with most of them during the summer season. We will include this information in the sample collection section which we have expanded (Lines 527 - 530).**

Some specific suggestions:

Line 78-81 The sentence would benefit from being shortened. That section is kept quite general. Therefore, I think it would be important to state that the definition of landscape functional connectivity can vary between different diseases vectors.

**We thank reviewer for the comment. We believe the definition of landscape connectivity is important for readers unfamiliar with landscape genetics/ecology. However, we have clarified landscape functional connectivity will vary for a given vector species in lines 142 - 147.**

Line 107 delete “explored”.

**We have deleted the word ‘explored’ (Line 112).**

Line 127 As mentioned in my general comments. Is the level of vector control the same across all sites? Are there other vector control interventions used in the area?

**We have already addressed this in the reviewer’s previous comment in lines 491 - 495**.

Line 130 It would be nice to expand a bit on other factors that might be linked to the landscape elevation parameter. What other factors are there? Does temperature seasonality affect the species?, Are there biannual variations in triatomine abundance at various landscape elevations?

**Again – we have already addressed this in the discussion in line with the reviewer’s previous comment in lines 771 - 796**.

Line 131 The authors suggest frequent and spatially targeted intervention. I agree, but it might be good to explain in a paragraph how the current vector control looks like.

**As above, we have already addressed this in lines 491 - 495.**

Line 133 Is this the specific recommendation for Loja or is this recommended for similar sites that show high gene flow and fragmented populations?

**We have amended to reflect broader applicability (Line 169)**.

Figure 1. Unfortunately, the resolution was too low to read the samples ID in Figure 1b. Potentially Figure 1 needs to be split into at least 2 Figures. It is not intuitive to look through the different parts of the Figure from right to left (a on the right, c on the left). I would prefer it the other way around.

**The image resolution has been resolved so the tree labels in Fig 1b can be easily read. We have re-ordered the images so they can be read more intuitively. We would prefer the keep the images in the same compound figure as it assists the viewer to interpret the genetic information in its geographic context. Figure specifications has been validated using the PACE tool to adhere to PLOS Genetics figures specifications.**

Line 372 Please clarify the first part of this sentence.

**We have made some modification to improve clarity (line 413).**

**Reviewer #2:** This paper reports a substantial body of work that adopts a landscape genomics approach to investigate the dispersal dynamics of Rhodnius ecuadoriensis in southern Ecuador. Studying vector dispersal dynamics and adaptation is certainly a relevant and topical research area. I really appreciated the effort of the authors in putting this study together, which includes a substantial number of samples from various collection sites, an appropriate bio-informatic pipeline to recover SNP genotypes and relatively challenging statistical analyses. I like the fact that authors included collection sites with both wild and domestic ecotypes, which reflects a correct study design for the aims outlined in this study. I also think that the results presented here, in particular those stemming from the landscape genomic analyses, are certainly worth publishing in a journal as PLoS Genetics.

My main concern is that the authors claim to have identified genomic regions linked to adaptation to the domestic setting. I think that this may be overstated because - in my opinion - the authors merely found some outlier loci between domestic and wild vector populations, without further evidence that these loci are truly adaptive. Based on the title alone, I truly expected to read substantial novel insights on the genes, gene arrays or genomic regions that are involved in the anthropogenic adaptation of this vector species. Unfortunately, there is merely a description of some outlier loci and the discussion barely touches on this topic. There is in fact only one SNP (LOCUS ID 15732) that was identified as an outlier SNP by all statistical analyses, but the location of this SNP in the genome remains unknown. This leaves the reader with nothing more than simply a bunch of SNPs identified by various programs without any clear discussion of their role or evidence that these are truly adaptive. I think that this problem could be simply solved if the authors are willing to downplay their title and some statements throughout the text (see below), and sell the landscape genomic results as the main outcome of their study.

**We have modified the title to ‘Population genomics and geographic dispersal in Chagas disease vectors: landscape drivers and evidence of adaptation to the domestic setting.’ to put less emphasis on the adaptation part. We have also modified elsewhere (Lines 39, 257, 266, 290, 422, (also 460 where we clearly discuss limitations)).**

My second main question is whether the authors have considered doing ADMIXTURE/STRUCTURE analyses or similar analyses that would allow to investigate population structure and potential hybrid/uncertain ancestry of these samples? I think this may be important to consider, as for the landscape genetic analyses the authors estimate genetic distance between collection sites, but without considering the potential that some of these collection sites may constitute a single population or a mix of populations. Some clarification is needed here.

**We appreciate reviewer’s concern about our criteria for using collection sites as the unit of our landscape mixed modelling analyses. There are a few problems here. The first is the dangerous circularity in assigning individuals to populations by genetics prior analysis of F_ST_. In doing so one can artificially inflate discontinuity or discontinuity between sites. To assist medical entomologists and the Chagas control program, we need to indicate the extent of geneflow between the sylvatic environment and domestic structures. Pre-sorting individuals by genetic population will cloud this issue.**

**Secondly, based on our hierarchical (F_ST_ 0.26), phylogenetic and DAPC analyses, we have strong evidence that collection site clustering is appropriate (Lines 199 - 217). Unlike STRUCTURE/Admixture, model-free approaches (like DAPC) do not rely on a particular model of divergence/admixture/mating structure and are flexible for inferring complex population structures (Jombart *et al.* 2010) such that of *R. ecuadoriensis* in Loja. The demography history of our populations likely deviates from STRUCTURE’s simple assumption of long divergence of *K* ancestral populations followed by a mixture phase.**

**Furthermore, DAPC provides some information about the inter-relatedness of clusters – which STRUCTURE does not. We deliberately chose DAPC for this reason.**

**Nevertheless, at reviewer suggestion we ran a STRUCTURE/Admixture analysis in which cross-validation plot suggests that *K* = 20 is the optimal cluster number to explain the ancestry of this population, closely related to (and congruent with) the total number of collection sites (n = 25). We have included admixture methods in lines 743 – 746 and admixture bar plot and cross-validation error plot in the supplementary results.**

My third main question is how the wild and domestic ecotypes were initially defined? In the methods section it is merely stated that a widespread spatial sampling of ecotypes was done. But how are these ecotypes defined? When is a bug classified as domestic and when as wild? This information is important given the aim of the study.

**We have expanded the sample collection and study area section to resolve this question. We have included ample references on the sampling design in the study area over the years which provides details on the larger CISeAL collection as well as our subset. As mentioned in lines 536 and 544, triatomines were defined as domestic when collected in the human built environment and wild when they were found inside wild animals nest > 3km away from domestic sites.**

Other concerns:
Page 7 - line 147. Not sure where to find S2 Table.

**We thank the reviewer for pointing out this typo. Here, we are referring to ‘S1 Table’ which will be provided as supplementary information (Line 187).**

Page 8 - lines 165-170. It is difficult to evaluate this because the map in Fig 1 doesn't show all collection sites. Also, if geography is the main factor impacting topology, why not colour accordingly instead of ecotype?

**Here our aim was to show the reader genomic differentiation in the collection sites for which both domestic and wild samples were collected. Including multiple collections sites for which only domestic or sylvatic samples were collected would not be helpful. We colour tree branches to show geography and tip labels to show ecotype and have updated the figure legend (line 219) to highlight this.**

Page 8 - line 172. You report low Fst between ecotypes. For comparative purposes it would be useful to also have Fst between collection sites.

**Collection sites differentiation is shown in Fig 4b. G_ST_ is a comparable measure to F_ST_ but corrects for sampling a limited number of individuals. To clarify, crude G_ST_ values are now shown in Supplementary data (Line 751). Importantly, in Figure 1 we also show a test for F_ST_ – so not only are the ecotype of pairwise comparisons low, the majority (5/7) are not statistically different from zero.**

Page 10. Here the authors repeatedly use the word adaptation / adaptive loci / signal of adaptation or alike, while there is no evidence at all that these loci are adaptive. Better to stick to outlier loci throughout, unless you have proof that these loci are linked to genes + you know the role of these genes + you have evidence that these genes play a role in domestication (e.g. through knock-out etc.)?

**We have replaced those words with outlier loci, or ‘possibly adaptive’ through-out the manuscript.**

Page 11 - line 230. Did the authors try a BLAST approach of a consensus read covering locus ID 15732?

**Yes, we BLAST searched consensus sequence of locus ID 15732 but unfortunately there was not hit on *R. prolixus* draft genome and *R. ecuadoriensis* genome is not available (Line 299).**

Page 12 - line 254-264. This paragraph is somewhat confusing. First the authors state that only one SNP from the RDA analysis is mapped to the Kruppel gene. Then later in the paragraph the authors state that there are three mapped loci under balancing selection from the fsthet analysis. This is the first time the authors describe balancing selection or the fact that they found loci under balancing selection. In the previous paragraphs they are always referring to outlier loci (so called adaptive loci). More-over, one or a few of these three SNPs under balancing selection also map to the Kruppel gene. Does this mean that there is one SNP from RDA analyses (and under so called adaptive selection) and one from fsthet analyses (and under so called balancing selection) in the same gene? This seems odd.

**We thank the reviewer for pointing out the confusion in these lines. SNPs are different between RDA and Fsthet analyses. There are not SNPs under balancing selection in any of the analysis and we have clarified fsthet SNPs are outlier loci (Line 292) with F_ST_ higher than the average neutral loci distribution as indicated in lines 258 -260 and 695. We apologise for the typo and confusion.**

Page 14 - line 300-301. I'm not sure that this statement is correct. Or at least it is not clear from Figure 4b. Sure, there is one obvious cluster (NJ, RT, HY, SJ and EX) and there is one obvious outlier (NT), but the other clusters seem less obvious. For instance, BM seems to be lowly differentiated from the cluster including CG, BR etc.

**We have modified this sentence to indicate that some sites were closely clustered and others not. (337)**

Page 15 - line 340-341. Even though bootstrapping clearly demonstrates that geographic distance may be a strong predictor, the author still claim that elevation is the strongest predictor based on results without bootstrapping. This seems like the authors are trying to deceive themselves or the readers. Based on these bootstrapping results, both distance and elevation are strong predictors, which also would make sense as vectors do not travel far.

**We have updated this section to reflect the uncertainty as well as the impact of our heterogenous sample collection distribution on the bootstrapping analysis. (376 – 378)**

Page 24 - line 529. For completion, please clarify -m, -M and -n to avoid forcing the reader to find this out themselves online.

**Thank you to the reviewer for suggestion this clarification on these assembly parameters. We agree we should facilitate this information to the reader and a comprehensive explanation of such parameters have been included in lines 587 – 594 and references are provided in the main manuscript as well as in the supplementary methods section.**

Figure 2b. There are loci on the right associated with wild ecotype. By the same logic, loci on the left are associated with domestic ecotypes? But the authors implicate both left and right loci are involved in adaptation to domestic ecotypes? Also, what is on the x-axis - how to interpret these values? Also here the authors refer to adaptive loci - better to stick with outlier loci.

**We have updated the figure 2 legend to clarify x-axis values (SNPs z-transformed loadings – methods line 690) represent the association strength to the constrained predictor (RDA 1), ecotype. We also clarify SNPs at the at ± 2 SD and ± 3 SD separated from the mean SNPs loading distribution of this axis are outlier loci showing a strong relationship with the predictor variable, ecotype (lines 275 - 278). Both are at some degree associated to domestication given the direction alongside RDA 1 axis shows if the allele frequency is positive or negatively associated to domestication. However, we agree QTLs and common-garden experiments would be necessary to identify domestic adaptations to the domestic niche as we discussed in limitations (Line 460)**

Figure 3b. Map of collection sites should come much earlier in the manuscript.

**We provide a map of selected sample sites in Figure 1 adjacent to the relevant analysis, and a full map of sample sites next to their relevant analysis in Figure 3. To do otherwise would confuse thing unnecessarily.**

There is a problem with the ordering of Figure and Table numbers. Also, often there is referral to the figures in S1 Methods, but many of these figures in S1 methods are results. It would make more sense to split these.

**Thank you for your helpful feedback and we agree splitting this way will help reader to link main figures and supplementary material. We have now corrected primary findings Figures and Tables, as well as supplementary Figures, Tables and Methods order throughout the manuscript. This now make it easier for the reader to look at the main findings and then refer to the supplementary information. As suggested, we have split Methods from supplementary Figures and Tables.**

The paper would benefit from some proof-reading. E.g. line 57 says populationS pairs; line 61 says landAcape genomic; line 72 says vector-BONE diseases, line 96 Alternately, etc. (I'm not going to list all of them here).

**We thank the reviewer for highlighting the above errors and we have undertaken a thorough proof-reading of the revised manuscript.**

**Reviewer #3:** This was a very comprehensive study using a range of methods to understand complex population structures among sylvatic and domestic vectors of Chagas disease. While I really enjoyed the paper, I found it difficult to extract the key findings from the results section and found some discrepancies between the aims and conclusions of the study. Specific comments below…

1. The abstract indicates the study focused on identifying adaptations that allow wild populations to enter the domestic environment however this is inaccurate. The focus is much more on understanding patterns of gene flow between wild and domestic sites

**We appreciate and thank the reviewer for their comment. This is in line with Reviewer 2’s comments above. We have made extensive edits in line with their comments that we hope will satisfy Reviewer 2. We consider our study focused on both topics and we have provided an appropriate methodology to evidence gene flow, identify SNPs associated to the domestic niche and ecological drivers of triatomine dispersal. However, we agree QTLs and common-garden experiments would be necessary to identify domestic adaptations to the domestic niche. We have made changes to the abstract to focus on both gene flow patterns and identify genomic associations to the domestic niche**

2. First paragraph makes some bold statements related to the research conducted here (modest changes drive epidemics) but there is little context provided that is relevant to this work and the links with second half of the paragraph (landscape heterogeneity) are weak. Would prefer to either have the authors expand on the first point with sufficient references, then introduce the second point later and in association with specific challenges for triatomine. As it reads the first paragraph is too broad

**We have expanded the first point ‘modest changes drive epidemics’ (Lines 71-81) with several refences, mainly in mosquitoes which are widely studied in that sense. We have also added a paragraph on what is known about in triatomines adaptations that may be involved in the domestic niche (Lines 117 - 132); however, most of what it is described is based on morphological and gene expression studies. This is an important gap in triatomine, and our study tried to resolve, and more importantly motivate genomics of domestic adaptation in triatomine. Overall, however, we feel that it is important to start ‘broad’ to highlight the relevance of the field.**

3. There isn’t sufficient background to understand triatomine population structures, and what is in volved in “domestication”. Do they enter the home seasonally? Would it be appropriate to elaborate here on what type of adaptions are required between the two environments? What does domestication involve in terms of changes in feeding/mating/oviposition/climate tolerance, etc?

**Thanks for your comment. We have elaborated on what changes may be required for a life in the domestic niche (lines 117 - 132) and we have expanded on triatomine population structures (lines 132 - 145) and mentioned house reinfestation a perennial risk in some regions but not others.**

4. Frame the research question in the context of what is known/unknown about the species. Why not highlight historical difficulties in controlling the populations with indoor insecticide? Previous studies have shown R. ecuadoriensis gene flow between sylvatic and domestic habitats and reinfestation after spraying, etc.

**We agree on framing the research question based on what we know/not know about this species. We have restructured the introduction to described triatomine reinfestation is the main impasse to vector control (lines 92 - 95), little is known about the genetic changes involved in adaptation to the domestic niche (lines 117 - 132) and ecological factors driving triatomine dispersal (lines 134 - 147). This background leads to state the problems in lines 154 - 159.**

5. Line 102, I’m unclear on the relevance of pigs as an example here. First the authors need to demonstrate clearly that sylvatic and domestic populations are genetically distinct (if that’s the case).

**Thank you for highlighting the confusion here. We agree this example is not relevant and we have included a more appropriate example within arthropods. We have used the honey bee in America as an example to show introgression between wild and domestic linages can lead to domestication and appearance of domestic traits. (Lines 103 - 107)**

6. Can the authors clarify when they describing domestication as a long-term evolutionary event or as an occasional occurrence where wild populations infest the home, these two points occasionally became confused throughout the manuscript

**We have clarified both terms in the manuscript. We refer as ‘domestication’ as long-term evolutionary sense and ‘colonisation’ of the domestic niche as infestation from wild to domestic ecotope (Line 100).**

7. The introduction would benefit from a clear problem statement earlier on about why these questions are relevant to Chagas control and triatomine populations. It appears in lines 121/122 but the background leading up to this lacks focus. Furthermore the aim as laid out (from line 124) doesn’t reflect the methods and results that follow.

**We really appreciate the reviewer comments and feedback on the introduction. As mentioned above, we have structured the introduction to give ample background and why our research questions matter for triatomine control. After that, we have summarised these problems (Lines 154 - 159) and we have frame our aims to reflect our methods and results (Line 160).**

Results

8. There are a number of instances where the primary result that is reported within a paragraph refers the reader to supplemental results. Since these should all be supplementary to the primary findings, I would suggest giving an overview of the data (numbers collected, etc) without sending the reader to the supplement, then presenting the primary findings with reference to figures, before presenting the supplemental results and referring the reader to consult the additional document.

**We have endeavoured to provide summaries of the relevant data without sending the reader to the supplementary data. (e.g. Line 536, 192 – 197, 199 – 217, 324, 339).** **We have also reordered all primary results as well as supplementary figures and tables to make it easier for the reader to refer to the supplementary results hyperlinks in the published manuscript.**

9. Line 161 – is it correct to call these two populations sympatric? Are they within each other’s dispersal range? Some studies have hypothesized that the sylvatic and domestic populations are isolated.

**Yes, they are within each other’s dispersal range. Populations are separated by up to 3 Km. We confirmed populations are not isolated as it occurs in other highly domesticated triatomines, e.g., *Rhodnius prolixus* and *Triatoma dimidiata*. This was presented in the introduction in lines 83 – 95.**

10. In terms of “Genetic loci correlated with domestic colonisation”, colonisation refers to population movement, in this case movement from a wild to domestic setting. I wonder if it’s possible to determine that the loci are association with migration/colonisation or are there other possibilities?

**We are looking at a genotype-by-environment interaction with multiple biological replicates. As such outliers we identify are likely to result from the influence of the ecotype.**

11. In general, results may be clearer if hypotheses were stated above

**We have modified the final paragraph of the introduction and hope the hypotheses being tested are now more clearly stated (160 – 172)**

12. Table 1 and 2 take up a lot of space but don’t convey much clear information on what parameters influence genetic differentiation. Is AIC that important here?

**We have reduced the number of columns in tables 1& 2 and more clearly stated the best supported models.**

13. Please clarify whether is domestic/sylvatic site locations are included as a variable alongside the landscape variables? What about the variable of time (since collections were over fourteen years)? Were individuals from the same households and nests sequenced, which may influence the sample size needed to address these questions. It seems there is a lot of variability that is not accounted for in the model. Without sufficient information in the methods about the collection locations, I can’t tell if the study is appropriately powered to determine landscape drivers of dispersal.

**We appreciate reviewer’s comments. We have expanded the collection sites in the sample collection and study area section in lines 520 – 544. As answered to Reviewer 1’s comments above, including parameters/variables such as history of vector control, domestic/sylvatic locations and time may be difficult in this type of models which use grids with values spanning the study area to estimate resistance to gene flow. In that sense, differences between domestic/wild niches may be captured by variables such as land cover type which we used to investigate the variation between fragmented (domestic environments, crops, etc) and natural areas. As reviewer comments, collection was carried out over a long period, however, our hierarchical analysis suggested collection sites subdivision is significantly stronger that ecotopes and time (Lines 213 – 217).**

Discussion

14. The summary at the beginning of the discussion is very helpful to distil key findings, I would suggest the other sections (abstract, statement of aims and research questions) mirror this.

**We have now modified the final paragraph of the introduction to state the hypotheses more clearly. Several modifications have also been made to the abstract.**

15. In the second paragraph bringing up the evolutionary processes that led to colonisation with examples taken from bloodfeeding in Culicidae, however this is tangential and the section would be stronger if it focused on triatomines

**We now focus initially on triatomines in the second paragraph, however we retain some discussion of other species as we feel it is vital to set our work in its broader context.**

16. The mapping of outlier loci to genes related to saliva production and embryonic development is mentioned in abstract (indicating it is a key finding) and discussion, but I don’t see it mentioned as a result. If this is a key finding it should appear in the results section first.

**These results are clearly shown in the results under the ‘Mapping outlier loci to the *Rhodnius prolixus* genome’ heading line 290.**

17. The study wasn’t designed to determine which genes enable domestication (requires QTL, etc) but there are a few instances where the authors are trying to stretch their rationale to identify genes identified are required for adaptation. Greater care should be taken to communicate the aims and the limits of the study.

**We have now ‘toned down’ reference to adaptation throughout the study in line with previous reviewer requests.**

18. There are attempts here to generalise findings to regional triatomine control and support for IVM which is warranted. Unclear how the conclusion to target secondary vector species arose from the study findings

***R. ecuadoriensis* is a secondary vector species – we have edited line 512 to clarify this.**

Methods

19. Info on broader study design is needed, how were houses/sites selected (either for the primary collection or for secondary analyses). How were “wild” triatomines targeted? What was the minimum/maximum distance between domestic and wild collection sites within a locale?

**Thank you for the helpful comment to improve study design. We have provided further references in the sample collection and study area section on the surveys and sampling design in Loja during over the years for the large triatomine collection as well as focusing on this subset. Houses/sites were selected by random sampling, and depending on the terrain accessibility, resources availability and community permission. We also provided information on the minimum/maximum distance between domestic and wild ecotopes. Lines 520 – 551.**

20. Section on GIS data includes a mix of methods, background and rationale, and possibly some results? (“absence of roads was a strong barrier”). Would prefer greater clarity in the methods, highlighting only what the authors have done with the data, and ensure background and limitations to the assumptions are covered elsewhere. Similarly the ResistanceGA provides background to published methods and could probably be trimmed down.

**We have made several edits and cuts in line with these suggestions (Line 796).**

21. In general I found the frequent cross referencing to the supplementary material a distraction, so please ensure the most salient information is available in the text

**We have made some edits in line with similar suggestions made by the reviewer earlier on.**

22. Supplement figure 2, I found this pictorial overview of the methods really useful, is there space to include in the main paper?

**We have moved this figure into the main body of the manuscript within the Methods section where we described estimating gene flow with resistance in line 747.**

Minor edits

Abstract

31 – use synonym for “man-made” (the built environment, domestic, human habitation, etc)

**We thank the reviewer for noting this word and suggesting a more appropriate alternative (e.g., lines 32 and 56).**

32 – In the second part of the sentence “their dispersal” would refer to adaptations rather than vectors. Consider rephrasing for clarity.

**We have clarified dispersal refers to vectors rather than adaptations (line 31).**

Summary

57 – change to “population pairs”.

**– Thank you, we have changed this word to ‘population pairs (Line 55).**

59 – misspelling of hindered.

**– We have corrected this typo (Line 60).**

60 – change to “triatomines”.

**– Triatomines has been added to this sentence (Line 60).**

Introduction

Line 97 Is domestication the correct term to use for zoonotic parasites?

**We have clarified domestication in vectors and zoonotic parasites are associated to the process (Line 99).**

132 – disease should be lower case.

**– We have changed disease to lower case (Line 170).**

134 – “genomic basis”.

**– This word has been corrected (Line 171)**.

Results

Line 316 “selecting”.

**– Thank you for indicating this error. It has been corrected (Line 354).**

Line 329 “than”.

**– The typo has been corrected (Line 366).**

Fig 1. Panel 1A is in the lower right, and would be clearer if ABC were positioned left to right.

**As also suggested by Reviewer 1, we have position panels from left to right and improved clarity in panel b. We have enlarged the phylogenetic tree and sharpen the labels. We checked figures journal specifications with PACE tool.**

Fig 1b is illegible.

**– We have modified as above.**

Fig 3b the site names and colours are difficult to discern. Similarly for 4a.

**As above, Figure clarity in Figure 3b and 4a has been improved by increasing the sharpness, size and colour contrast. We have checked all figures with PACE tool to adhere to journal figures specifications.**

Discussion

Line 415 “significance of such a change”.

**– We have corrected this part of the sentence (Line 457).**

Line 432 remove extra comma before “are”.

**– Thank you, we have removed the comma (Line 474).**

Line 436 – again, references to mosquitoes is a distraction

– **We disagree – important to keep things broad**

Line 437, additional context needed about this study on parasite isolates.

**– Additional context has been included from this study (Line 480).**

Methods

Line 498 should read (443/502).

**– We have corrected this error (Line 555).**

Line 520 remove “reads”.

**– The word has been removed from this sentence (576).**

Line 524 “yielded”.

**– It has been corrected (580).**
