# Supplementary material for: Population genomics and geographic dispersal in Chagas disease vectors: Landscape drivers and evidence of possible adaptation to the domestic setting
Source: PLoS Genet. 2022 Feb 4;18(2):e1010019. doi: 10.1371/journal.pgen.1010019 (PMC8849464; doi:10.1371/journal.pgen.1010019)
Supplement: Attachment — Submitted filename: Response_to_Reviewers.docx [file pgen.1010019.s024.docx]

University of Edinburgh,

The Roslin Institute,

Easter Bush Campus,

Midlothian,

EH25 9RG

Scotland

5^th^ January 2022

**Re: Revision of PLOS Genetics PGENETICS-D-21-00937R1**

To whom it may concern,

We are grateful with editors and reviewers for your valuable time and careful consideration of our manuscript which we believe is an important and highly regarded topic within the Chagas disease and arthropod-borne diseases research community. We have now made further modifications to the manuscript and all comments have been carefully addressed. Our thanks to the reviewers - their feedback has been highly constructive. We note that two reviewers of our previous draft were largely happy with our changes / responses. In this most recent revision, we have attempted to satisfy the final reviewer, reviewer 3.

Our responses to reviewers are detailed below (bold purple) with a description of the changes made in the manuscript.

We greatly appreciated the time taken in considering our manuscript and would be available to address any further questions should they arise.

Yours faithfully,

Luis Enrique Hernandez Castro

Mobile: +447452992678

Research Fellow – Centre for Tropical Livestock Genetics and Health.

University of Edinburgh

Reviewer's Responses to Questions

**Comments to the Authors:
Please note here if the review is uploaded as an attachment.**

Reviewer #1: In general the authors have sufficiently addressed the points I raised. There are just a few minor points.

The explanation in the authors response regarding the rather broad spatial context of existing studies that investigated triatomine distribution was fine, but unfortunately, the description in the manuscript is less clear (what are "broad" and "detailed" vector population dynamics).

**As reviewer comments, our description in the introduction was less clear than our response to reviewer. We have now expanded and clarify accordingly to our response in previous review round. We referred to ‘broad’ as broad spatial distribution and ‘detailed’ to local population dynamics such as movement. We thank the reviewer for pointing this out which has now been corrected in lines 141 - 142.**

In the method section, in line 528, the authors explained that the sampling was done "mainly" during the summer. As mentioned by the authors the number of triatomes can vary throughout the seasons and the definition of "mainly" can be anything between 51-99%. I would therefore suggest to be more specific.

**We apologise with the reviewer if our sampling response was not specific. We acknowledged in previous revision round seasonal variation on triatomine abundance is expected; however, their presence is constant throughout the year. We do not expect seasonal variation to have an effect in our models which rely on the numbers and high-resolution genetic markers rather than the presence (counts) of the triatomines in the zone. Perhaps, Poisson/logistic regression models (or other algorithms) for species distribution models may need adequate large triatomine counts to produce reliable predictions in which seasonal variation may have a larger impact. Nevertheless, we followed reviewer recommendation to be more specific about sampling in line 570.**

Finally, there are several unnecessary typos that were made in the updated sections and the paper would benefit from a repeated proof-reading.
For example: presence in stead of present (line 139), showed instead of shown (line 139), summe instead of summer (line 528).

**We have now re-proofread and amended the typographic errors.**

Reviewer #3: The introduction has been strengthened with more relevant background. I still find it a distraction to begin the introduction with so many mosquito references, however I will leave this one to the authors.

**Many thanks. We are happy with the broad scope of the first paragraph. Its aim is to highlight the importance of domiciliation of vector species. All the big papers are in mosquitos…**

The additional information on sample collection is clear. The inclusion of Fig 5 is good, however it would be more informative if it appeared before the results. IS it possible to first reference the figure when the study design is presented?

**As the reviewer suggested, we have placed Fig 5 (now Fig 1) before results section where we now introduced our framework and define landscape functional connectivity (Lines 143 - 152). We hope this is now more informative and in line with reviewer’s comments.**

The authors responded to many of my comments by saying paragraphs have been rewritten - for example to clarify the hypothesis in the end of the introduction, or to improve the description of GIS methods. However I found very few meaningful changes that were related to my criticisms.

**We are sorry to hear that edits were not perceived as meaningful on the first round of revision. We have made further changes.**

- **Background/rationale was included within the GIS section for easy availability to the reader and to argue for the use of these variables. However, we agreed for clarity is better place in the introduction where we describe triatomine structures, and the ecological factors that may be driving triatomine and *R. ecuadoriensis* dispersal in lines 147 - 152.**
- **We would like to clarify “absence of roads was a strong barrier” is not a result but how we reclassified roads data. We have rewritten these sentences to avoid confusion (Line 813).**
- **The ResistanceGA section was trimmed down since the first revision round (Lines 821 – 830).**
- **We have also added a summary at the end of the introduction (Lines 177 – 180) to better represent our research questions/hypotheses. These reflect the background we introduced on triatomines structures, possible adaptations to the domestic niche and ecological factors driving their dispersal. Then, we laid our aims (Lines 182 – 185) and provided a summary of key findings mirroring the beginning of the discussion as suggested by the reviewer on the first revision round (Lines 185 – 204). We hope the changes to these sections are meaningful and reflect the results and methods that follow.**

Previous comments highlighted the need to improve the figures, and this is still the case. Many of them are still illegible.

**We thank reviewer for pointing out this issue. Original figures files have been improved since the first round of revision as reviewers pointed out. We passed the figures on the journal PACE tools as advised during the submission process and we modified the sharpness and size of most of the figures, together with text. We have confirmed with the editorial office (correspondence on 30^th^ December 2021) legibility issues are caused in compiled PDF file during compression during the submission process; however, they confirmed original figures are accessible for reviewers for evaluation. Those original figures files will be used as source for publication and not from the compiled PDF file.**

The reviewers raised some limitations of the study and the authors have responded by softening some of the language around adaptation. This is the bare minimum and I think the paper still needs to clearly acknowledge the limitations of the study (not just related to genome coverage).

**In the first revision round we toned down the manuscript title and all sections in the manuscript that mention adaptation. We also go into considerable detail on potential improvements. In the discussion, for example, we clearly state that QTL and GWAS will be necessary to fully understand the genomic processes of adaptation.**

**We have, nonetheless, expanded our statement in the discussion to highlight the limitations of the study and possible future work to improve biological inference (Lines 496 - 504) We have also recommended further research in triatomines genomics in the light of these limitations (Lines 508 – 512 and 551 - 554). We hope these statements satisfy reviewer comments.**

The newly written sections should be carefully proofread, I noticed quite a few examples of spelling and grammatical errors

**As assured above to reviewer 1, we have revised thoroughly our manuscript for errors, especially the newly added sections. We thank the reviewer for highlight these spelling errors.**
